# New Insight into the Multi-Scale Structure and Anti-Digestibility of Nano-Scale Amylopectin Ternary Assemblies Prepared Under High-Power Ultrasound

**DOI:** 10.3390/foods15061021

**Published:** 2026-03-14

**Authors:** Bo Li, Yanjun Zhang, Zuohua Xie, Lixiang Zhou, Yanru Zhou, Xin Yang, Weihong Lu

**Affiliations:** 1 School of Medicine and Health, Harbin Institute of Technology, Harbin 150001, China; 13654529477@163.com; 2 Key Laboratory of Processing Suitability and Quality Control of the Special Tropical Crops of Hainan Province, National Center of Important Tropical Crops Engineering and Technology Research, Spice and Beverage Research Institute, Chinese Academy of Tropical Agricultural Sciences, Wanning 571533, China; 3 Chinese Academy of Tropical Agricultural Sciences, Haikou 570105, China; 4 Jiangxi Deshang Technology Group Co., Ltd., Zhangshu 331299, China; xzh10030606@163.com (Z.X.);; 5 Jiangxi Deshang Pharmaceutical Co., Ltd., Zhangshu 331299, China

**Keywords:** high-power ultrasound during self-assembly, nano-scale amylopectin ternary assemblies, *Euryale ferox* amylopectin, multi-scale assembly structure, anti-digestibility mechanism, waxy starch

## Abstract

High-power ultrasound has been widely used to regulate the anti-digestibility of starch-based products, including the formation of resistant starch (RS-V) in amylopectin assemblies. This can contribute to the attenuation of postprandial hyperglycemia. However, the mechanisms by which high-power ultrasound modulates RS-V remain to be elucidated. Therefore, nano-scale *Euryale ferox* amylopectin (EFA) ternary assemblies were constructed under high-power ultrasound. All EFA assemblies exhibited ternary self-assembly peaks and V-type crystallinity. Combined chemometric analyses revealed that, with increasing ultrasound power, the rising self-assembly sites within B2 and C chains promoted the increase in self-assembly index but decreased semicrystalline lamellae thickness and structural fractal dimension. Consequently, a compact and ordered molecular cross-linking network was formed, contributing to increases in residual crystallinity, molecular weight, short-range order, and molecular density. This resulted in the shrinkage of digestion channel structures and optimization of the molecular gel network. As a result, the reduction in hydrolysis sites with increasing ultrasound power led to increased RS-V content (22.66–60.17%), causing a decline in the estimated glycemic index. The EFA–lauric acid–lactoglobulin assemblies prepared under 600 W ultrasound were the optimal composition and exhibited enhanced anti-digestibility relative to amylopectin assemblies derived from staple crops such as white waxy maize. The present investigation not only serves as a valuable supplement for studying the precise regulation mechanisms of nano-scale amylopectin RS-V, but also provides critical theoretical guidance for the development of foods aimed at preventing hyperglycemia.

## 1. Introduction

Hyperglycemia is a typical pathological manifestation of disordered glucose metabolism. Persistent hyperglycemia can cause various complications [1]. Excessive consumption of high-calorie foods, irregular lifestyle habits, and detrimental behaviors such as smoking and alcohol abuse are known to induce glucose metabolic disorders, and individuals exhibiting such habits are considered to be at high risk for hyperglycemia [1]. Resistant starch (RS), a unique class of starch that resists digestion and absorption in the small intestine within 120 min of ingestion [2], has gained attention as an important dietary component due to its ability to attenuate postprandial blood glucose elevation [3], enhance satiety [4], stimulate insulin secretion [5], and promote the proliferation of beneficial gut microbiota [6]. Precision nutrition has been defined as a targeted nutritional strategy aimed at modulating postprandial glycemic responses in populations at high metabolic risk, with structural regulation serving as a supporting technological approach. Therefore, RS can serve as part of an effective precision nutritional strategy for regulating dietary composition and controlling blood glucose levels, offering a promising approach for the prevention of glucose metabolism disorders.

After thermal processing of starch-based food materials, starch can spontaneously form enzyme-resistant crystalline structures through noncovalent interactions with lipids or proteins during the coagulation–precipitation process [7]. This self-assembly behavior results in the formation of nano-scale type-V resistant starch (RS-V) assemblies [8,9]. Compared with RS-I (physically entrapped RS), RS-II (native granular RS), RS-III (retrograded RS), and RS-IV (chemically modified or graft-copolymerized RS), RS-V exhibits a more enzyme-resistant and thermally stable, highly ordered multilevel architecture [7,10]. During the formation of ternary assemblies, the polar head and tail groups of proteins induce hyperconjugated electrostatic effects [11], thereby strengthening and broadening the noncovalent binding forces among starch chains, lipid chains, and the amide II regions of proteins [12]. As a result, a more compact and ordered supramolecular structure is formed [13] and the self-assembly index (SI) markedly increases [14]. Zhou et al. [6] defined the SI as the proportion of nano-scale assembled structures within the particle architecture. As a result, the content of RS-V in ternary assemblies is significantly higher than that in binary assemblies. In addition, amylose—which predominantly exists in amorphous regions—can be rapidly hydrolyzed by starch glucosidase and α-amylase, whereas amylopectin—mainly distributed in crystalline regions—can hinder the penetration of digestive enzymes [15]. Therefore, assembling the cross-lamellar long chains of amylopectin at enzymatic cleavage sites with fatty acids and proteins can generate more RS-V than amylose assemblies. However, although starches from staple crops such as maize, rice, and wheat generally contain a high proportion of amylopectin, it is characterized by a markedly shorter average chain length, a lower abundance of free side chains and trans-lamellar chains, and a reduced weight-average molecular weight (*M_w_*) [16,17]. These characteristics restrict the formation of RS-V assemblies [18]. Consequently, reports on RS-V_3_ formation from the amylopectin assemblies of staple crops are scarce. In contrast, previous studies have demonstrated that Euryale ferox amylopectin contains longer debranched chain length distributions, higher contents of free side chains and trans-lamellar chains, and greater *M_w_* when compared with staple crops [19,20,21,22]. Accordingly, *Euryale ferox* amylopectin (EFA) was selected as the raw material for the construction of a novel nano-scale ternary assembly. Moreover, fatty acids with relatively short carbon chains and low degrees of unsaturation, such as lauric acid (LA), when associated with proteins possessing high isoelectric points such as β-lactoglobulin (βLG), exhibited an enhanced negative surface charge, which markedly improved their emulsifying capability [2]. Consequently, it was suggested that the EFA assembled with LA and βLG might exhibit a higher SI together with an increased proportion of RS-V. Therefore, EFA–LA–β-lactoglobulin (βLG) was selected as the starting material for this study.

Existing research on improving the anti-digestive properties of starch ternary complexes has mainly focused on amylose modification, including modifying the degree of polymerization (DP) of chains [13], adjusting the amylose content [23], or enhancing chain extensibility [18]. Studies on amylopectin ternary assemblies have mainly focused on the digestive and physicochemical properties of different types of amylopectin ternary assemblies, while only a limited number of reports have attempted to improve their digestibility through enzymatic modification [14,24]. Compared with previous approaches used to prepare starch ternary assemblies, including homogenization, rapid viscosity analysis, and extrusion [25,26,27,28], high-power ultrasound technology (140–700 W, 20 kHz, 30–60 min) may represent an effective approach that could generate cavitation effects, promote molecular motion, and provide a suitable energy-level range to potentially prevent starch chain breakage, thereby enabling the construction of ternary assemblies with higher RS-V content [29,30,31]. Moreover, previous studies have shown that relatively low ultrasound powers (<200 W) generally provide insufficient cavitation intensity to effectively promote molecular rearrangement and complex formation, whereas excessively high powers (≥700 W) are more likely to induce starch chain scission and structural disruption due to strong mechanical and thermal effects [17,29,31]. Therefore, the range of 200–600 W was selected as a practical and widely accepted energy window that ensures effective cavitation-driven assembly while minimizing the risk of amylopectin chain degradation.

Overall, this study aimed to utilize high-power ultrasound during self-assembly to regulate cavitation and shear effects, relax EFA chain segments, and alter the availability and distribution of assembly sites. This was intended to efficiently enhance the self-assembly index (SI), internal interaction forces, structural order, and compactness of the formed EFA ternary assemblies, while reducing hydrolysis sites, modifying hydrolysis pathways, and ultimately increasing RS-V content. Furthermore, this research verified whether high-power ultrasound applied during the self-assembly process influenced anti-digestibility and the estimated glycemic index (EGI), which was used as an indicator to evaluate the potential postprandial glycemic response.

Therefore, for this investigation, nano-scale EFA–LA–βLG assemblies were first prepared using a temperature-controlled magnetic stirrer coupled with an ultrasonic cell disruptor. High-power ultrasound conditions during self-assembly were used to control the formation of assembly bonds. Furthermore, the dynamic changes in the distribution of assembly sites and the evolution of multi-scale spatial configuration and conformation structures were characterized by changes in ultrasound conditions. The RS-V content, digestion kinetics, and EGI were evaluated. Ultimately, chemometric analysis was employed to establish the relationship between supramolecular structure and anti-digestibility, thereby revealing the effects of high-power ultrasound on the digestibility mechanisms of nano-scale EFA ternary assemblies. In addition, waxy maize, waxy potato, waxy wheat, and waxy rice assemblies were obtained at an ultrasonic power of 600 W, reaching their maximum RS-V contents, and analyzed as supplementary controls to highlight the superior digestive application potential of the EFA assemblies. Ultimately, the research objective was to gain insight into the influence of high-power ultrasound during self-assembly on the multi-scale structure and anti-digestibility of nano-scale amylopectin ternary assemblies. This study not only supplements the current understanding of the formation mechanisms of nano-scale amylopectin with high RS-V content, which could more effectively regulate blood glucose levels while simultaneously supplying essential macronutrients, but also contributes to the high-value utilization of EFA products.

## 2. Materials and Methods

### 2.1. Materials

Purified Euryale ferox starch (98.79%) was sourced from Jiangxi Deshang Technology Group Co., Ltd., Zhangshu, China and the Spice and Beverage Research Institute, Chinese Academy of Tropical Agricultural Sciences, Wanning, China. Amyloglucosidase (3300 U/mL), glucose oxidase–peroxidase (1000 U/mg), and α-amylase (100,000 U/g) were obtained from Megazyme Ltd. (Bray, Ireland). White waxy maize, waxy potato, waxy wheat, and waxy rice were sourced from Jiangxi Deshang Technology Group Co., Ltd. and the Spice and Beverage Research Institute, Chinese Academy of Tropic Agricultural Sciences.

### 2.2. Separation of Amylopectin

The separation of amylopectin was performed based on the distinct solubility and sedimentation behaviors of amylose and amylopectin after complete starch gelatinization. Briefly, *Euryale ferox* starch was dispersed at a concentration of 3% (*w*/*v*) and fully gelatinized at 100 °C for 1 h to disrupt the native crystalline structure. The gelatinized slurry was then subjected to differential centrifugation (7000× *g*, 10 min), during which amylopectin was recovered as the precipitate, while amylose remained predominantly in the supernatant. The collected precipitate (crude amylopectin) was subsequently treated with 80% (*v*/*v*) methanol and vortexed for 20 min to further remove residual amylose, taking advantage of the higher solubility of amylose in alcohol–water mixtures compared with amylopectin. After methanol treatment, the suspension was centrifuged again (7000× *g*, 10 min), and the resulting precipitate was collected as high-purity amylopectin. The recovered amylopectin was finally ground and dried at 60 °C until the moisture content fell below 8% [24]. The isolated EFA exhibited a 98.22% amylopectin recovery, as determined via Lugol’s solution method, and similar amylopectin chain-length distribution results were observed for raw Euryale ferox starch and EFA (Table S1). These findings further suggest that this method achieved high-purity amylopectin isolation while maintaining the molecular integrity of the amylopectin chains.

The commercial amylopectin forms of white waxy maize, waxy potato, waxy wheat, and waxy rice starch were isolated using the other approach. Powdered material from commercial crops was mixed with deionized water (1:3 *w*/*w*) and processed using a colloid grinder to obtain a starch suspension. The dispersion was then passed through a 200-mesh sieve, and the filtrate was subjected to centrifugation at 6000× *g* for 10 min. The resulting sediment was subsequently treated with 0.5 M sodium thiosulfate (1:1 *w*/*w*) for 18 h, followed by centrifugation at 5000× *g* for 10 min, during which the dark-colored supernatant was carefully removed. The obtained amylopectin was washed three times with 50% (*v*/*v*) ethanol and dried at 50 °C for 24 h. The final product contained 99.15% amylopectin [14].

### 2.3. Construction of Nano-Scale EFA Ternary Assemblies

#### 2.3.1. Construction of Amylopectin Ternary Assemblies with the Ultrasonic Process

The EFA–LA–βLG assemblies were prepared using a temperature-controlled magnetic stirrer (RCT 5, IKA-Werke GmbH, Staufen, Germany) coupled with an ultrasonic processor (700 W, Cole-Parmer Instruments, Vernon Hills, IL, USA). A mixture containing 4 g of EFA, 200 mg of LA, 400 mg of βLG, and 50 mL of distilled water was introduced into the vessel. The suspension was first stirred at 960 rpm for 10 s, followed by continuous stirring at 160 rpm throughout the experiment. The temperature was increased to 50 °C for 1 min and then to 95 °C for 20 min. During the entire self-assembly process, the ultrasonic power was adjusted sequentially to 200, 300, 400, 500, and 600 W (20 kHz). Afterward, the system was cooled to 25 °C and maintained for 10 min. The obtained assemblies were washed with 50% ethanol and dried at 60 °C until the moisture content fell below 8% [12]. The EFA–LA–βLG assemblies prepared under different ultrasound powers were named U_200_ EFA assemblies, U_300_ EFA assemblies, U_400_ EFA assemblies, U_500_ EFA assemblies, and U_600_ EFA assemblies. All experiments at different ultrasound power levels were independently repeated five times.

#### 2.3.2. Preparation of Nano-Scale Amylopectin Ternary Assemblies

EFA assembly samples (5.0 g) were placed into a milling jar together with 50.0 g of zirconia balls with diameters ranging from 0.3 to 0.5 mm. The mixture was then processed in a ball mill (Simoloyer CM-01, Zoz GmbH, Wenden, Germany) at 1800 rpm for 60 min in an intermittent mode consisting of 5 min of milling followed by a 2 min pause. This procedure was applied to reduce particle aggregation and decrease particle size [32]. The amylopectin ternary assemblies appeared as a white powder.

### 2.4. Self-Assembly Properties

#### 2.4.1. SI Analysis

EFA assembly samples were mixed with ethanol and 0.9 M NaOH at a volume ratio of 1:1:9 and heated in a boiling water bath until complete dissolution was achieved. After the solution was cooled to 25 °C, it was diluted tenfold. An aliquot of 2.5 mL of the diluted solution was then brought to a final volume of 50 mL. Subsequently, 1 mL of Lugol’s iodine solution (saturated I_2_–KI solution) was added, and the mixture was allowed to react for 20 min for color development. The absorbance was measured at 548 nm using a UV–Vis spectrophotometer (UV-2700, Shimadzu Corporation, Kyoto, Japan), and the amylopectin content was determined from the standard curve [26]. The SI was calculated as follows:

(1) $$SI(\%)=100\times \left({Absorbance}_{native\;starch}-{Absorbance}_{comnplexes}\right)/{Absorbance}_{native\;starch}$$

#### 2.4.2. 1D and 2D Infrared Spectrum Analysis

Under continuous infrared drying lamp irradiation, 3 mg of starch powder was thoroughly mixed with 300 mg of spectroscopic-grade KBr using an agate mortar for 3–5 min under low-humidity conditions. The homogeneous mixture was then compressed into a 13 mm pellet using a manual pellet press. The prepared pellet was stored in a desiccator prior to Fourier Transform infrared spectroscopy (FTIR) analysis. The formation of EFA ternary assemblies was confirmed based on 2D FTIR (Nicolet iS50, Thermo Fisher Scientific, Waltham, MA, USA) analysis. A Hilbert transform algorithm was applied to analyze the characteristic peaks of the assemblies within the range of 4000–400 cm^−1^ in two-dimensional correlation spectroscopy (2D-COS) FTIR spectra. Moreover, a baseline correction was applied to the full FTIR spectra. Subsequently, the 1200–800 cm^−1^ region was extracted for Fourier self-deconvolution analysis. The short-range order of samples was quantified using the 1047/1022 cm^−1^ intensity ratio derived from the deconvoluted spectra [33].

#### 2.4.3. Crystalline Structure

The crystalline structure of the EFA ternary assemblies was characterized using an X-ray diffractometer (XRD, D8 Advance, Bruker AXS GmbH, Karlsruhe, Germany) equipped with Cu Kα radiation and operated at 40 kV and 40 mA. Prior to measurement, the freeze-dried starch powder was packed into the sample holder and carefully leveled to ensure a flat surface. Diffraction patterns were collected over a 2θ range of 4–40° with a step size of 0.02° and a scanning rate of 4°/min. The residual crystallinity (*R_c_*) and crystalline type were determined using the JADE 6.5 software [13].

#### 2.4.4. Particle Size Distribution

A nanoparticle size analyzer (ZS90, Malvern Instruments Ltd., Malvern, UK) was employed to determine the particle size distribution of the EFA ternary assemblies, which were fully dispersed in distilled water (1:10, *w*/*w*) with the assistance of ultrasonication at 200 W [34]. All experiments were independently repeated five times. The particle size distributions researched in this study were intensity-weighted hydrodynamic diameter distributions.

### 2.5. Multi-Scale Assembly Structure of EFA Ternary Assemblies

#### 2.5.1. Chain Length Distributions

The distribution of chain lengths within the EFA ternary assemblies was characterized via high-performance anion-exchange chromatography (HP-AEC) using a Dionex ICS-5000 system (Dionex Corporation, Sunnyvale, CA, USA). For enzymatic debranching, 10 µL of pullulanase (1000 NPUN/g, Sigma-Aldrich, St. Louis, MO, USA) was added to 5 mL of a 0.1% (*w*/*v*) starch dispersion adjusted to pH = 6, and the reaction was maintained at 60 °C for 24 h. Chromatographic separation was performed on a CarboPac PA200 column (3 mm × 250 mm; Dionex Corporation, Sunnyvale, CA, USA) operated at a flow rate of 0.4 mL/min [24].

#### 2.5.2. Semicrystalline Lamella Characterization

The semicrystalline thicknesses (*d*), amorphous thicknesses (*d_a_*), and crystalline thicknesses (*d_c_*) were calculated by observing the 1D scattering peaks obtained from a small-angle X-ray scattering instrument (SAXS, NanoSTAR, Bruker AXS Inc., Madison, WI, USA) [35]. Overall, 200 mg of EFA ternary assemblies was mixed with 4 mL of distilled water and subjected to centrifugation (6000× *g*, 15 min). The supernatant was analyzed by evaluating the 2D patterns of the SAXS at 50 kV, 30 W, and 1.5418 Å wavelength. The scanning range was set as 0.010 < *q* < 0.25 Å^−1^. The 2D patterns were then converted into 1D scattering curves based on the following Equation (2).

(2) $$L\left(r\right)=\frac{\int_{0}^{\infty }I\left(q\right)q^{2}\cos\left(qr\right)dq}{\int_{0}^{\infty }I\left(q\right)q^{2}dq}$$

where *r*, *q*, and *I*(*q*) represent the scattering space distance, scattering vector, and scattering intensity, respectively. The surface fractal dimension (*α*) was calculated according to $I(q)\sim q^{a}$. The mass fractal dimension (*D_m_*) was calculated using Equation (3).

(3) Dm=−α (−3<α<−1)

The average distance between crystalline and amorphous lamellae (*ξ*) and the thickness of crystallite units (*ξ_c_*) were calculated using Equations (4) and (5):

(4) $$\xi =Rc\times d_{a}$$

(5) $$\xi_{c}=\frac{\xi }{1-Rc}$$

#### 2.5.3. Molecular Configuration and Conformation

EFA ternary assemblies (10 mg) were mixed with 5 mL of 50 mM DMSO containing LiBr (2 mg/mL) and maintained at 90 °C for 24 h. After the solution was cooled to 25 °C, it was diluted with the mobile phase (50 mM DMSO/LiBr) and analyzed using a size-exclusion chromatography system (SEC, Wyatt Technology Corporation, Santa Barbara, CA, USA) equipped with parallel laser light scattering (LS) and refractive index (RI) detectors. Chromatographic separation (100 μL of sample) was carried out on Shodex OHpak SB-803, SB-804, and SB-805 HQ Phenogel columns (Showa Denko, Tokyo, Japan) operated at 60 °C and a flow rate of 0.3 mL/min. The ASTRA v7.3 software was used to analyze the molecular configuration and conformation parameters [13].

#### 2.5.4. Nano-Scale Surface Texture Characteristic

A total of 20 mg of the sample was fully dispersed in 100 mL of 50% ethanol, and a drop of the dispersion was deposited onto a single-sided polished silicon wafer. After solvent evaporation under ambient conditions, the silicon substrate was mounted on the AFM sample stage for measurement. EFA ternary assembly samples were scanned over a 2 μm × 2 μm area in tapping mode using an atomic force microscope (AFM, 5100 N; Hitachi, Tokyo, Japan) to obtain phase images, modulus maps, and 3D surface topography. The embedded software was used to calculate the root mean square roughness (*R_q_*), which reflected the characteristic size of the “blocklet” protrusion structures, as well as additional nano-scale surface texture parameters [36].

#### 2.5.5. Micromorphology Analysis

The morphology of the EFA ternary assemblies was examined using a field emission scanning electron microscope (FE-SEM SU8220; Hitachi, Japan) at an accelerating voltage of 15 kV and a magnification of 30,000× [37].

### 2.6. Thermal Gelatinization Properties

EFA ternary assemblies (2 mg) were mixed with 6 μL of distilled water in a liquid crucible and equilibrated for 24 h. Differential scanning calorimetry (DSC 2500, TA Instruments, New Castle, DE, USA) was used to determine the gelatinization enthalpy (Δ*H_g_*), peak temperature (*T_p_*), onset temperature (*T_o_*), conclusion temperature (*T_c_*), and gelatinization temperature range (*R*) over a temperature range of 10–103 °C at a heating rate of 10 °C/min [9].

### 2.7. Viscous Characteristics

Three grams of the EFA ternary assemblies was fully dispersed in 25 g of water, and the viscous characteristics of the EFA ternary assemblies were automatically measured using a rapid visco analyzer (RVA; Super 4, Newport Scientific Pty. Ltd., Warriewood, NSW, Australia) under the Standard 1 program. The recorded parameters included peak viscosity (PV), trough viscosity (TV), breakdown viscosity (BDV), final viscosity (FV), setback viscosity (SBV), and pasting temperature (PT) [38].

### 2.8. Characterization of Anti-Digestibility

#### 2.8.1. Digestive Fraction

One gram of the EFA ternary assemblies was completely suspended in 10 mL of 0.1 M acetate buffer (pH = 5.2). Then, 10 mL of α-amylase (3800 U/mL), amyloglucosidase (13 U/mL), and invertase (190 U/mL) were added to the acetate buffer, which was used to carry out the digestive fraction analysis (37 °C, 180 rpm). An aliquot of 0.5 mL of the digestion mixture was withdrawn, and enzymatic activity was completely terminated at 20 and 120 min through the addition of 70% ethanol, thereby ending the digestion experiment. The glucose release equivalents after digestion were determined using the glucose oxidase–peroxidase (GOPOD) method [14]. Rapidly digestible starch (RDS), slowly digestible starch (SDS), and RS-V were analyzed using Formulas (6)–(8).

(6) $$RDS\left(\%\right)=\frac{\left(G_{20}-G_{F}\right)\times 0.9}{TS}$$

(7) $$SDS\left(\%\right)=\frac{\left(G_{120}-G_{20}\right)\times 0.9}{TS}$$

(8) $$RSV\left(\%\right)=\frac{\left[TS-\left(RDS+SDS\right)\right]}{TS}$$

In these formulas, *G*_20_ and *G*_120_ correspond to the amounts of glucose released at digestion times of 20 and 120 min, respectively.

#### 2.8.2. Digestive Kinetics and Estimated Glycemic Index (EGI)

EFA ternary assemblies (200 mg) were mixed with sodium acetate buffer (15 mL, 0.2 M, pH = 5.2). Digestive kinetics was initiated by adding 10 mL of a preparation containing α-amylase (290 U/mL) and amyloglucosidase (15 U/mL), and the system was maintained at 37 °C under agitation at 150 rpm. During digestion, 0.5 mL of the reaction supernatant was collected at 0, 10, 20, 30,40, 50, 60, 90, 120, 150, and 180 min and immediately combined with 4.5 mL of absolute ethanol to stop the digestive kinetics. The glucose release equivalents were determined using the GOPOD method. The hydrolyzed percentage (*C*) was calculated according to Equation (9). The equilibrium concentration (*C_∞_*) and digestive rate constant (*k*) were subsequently analyzed using Equation (10) [37].

(9) $$Percentage\;of\;hydrolyzed\;starch\;\left(\%\right)=\frac{\Delta A(Samples)}{\Delta A(Glucose\;Standard)}\times 25\times \frac{100\%}{200\;mg}\times \frac{162}{180}$$

(10) $$C=C_{f\infty }\left(1-e^{-kt}\right),\;C_{f\infty }\leq 100\%$$

In this equation, ΔA denotes the measured absorbance. The value 25 accounted for the dilution applied to the 0.5 mL reaction supernatant, while the ratio 162/180 was used as the stoichiometric coefficient for converting starch equivalents into glucose. In addition, AUC refers to the integrated area beneath the digestion curve. Based on these parameters, the hydrolysis index (HI) and the estimated glycemic index (EGI) were determined using the equations provided below.

(11) $$AUC=C_{f\infty }\left(t_{f}-t_{o}\right)-\left(\frac{C_{f\infty }}{k}\right)\left[1-exp-k\left(t_{f}-t_{0}\right)\right]$$

(12) $$HI=\frac{AUC\;(sample)}{AUC\;(white\;bread)}$$

(13) EGI=39.71+(0.549×HI)

in which the *AUC* for white bread is 210.1 and the hydrolysis index HI is typically 100 [4].

### 2.9. Statistical Analysis

Statistical analyses, including ANOVA, 2D principal component analysis (PCA), and the determination of average values and associated variability, were carried out with SPSS Statistics version 22.0 (IBM Corp., Armonk, NY, USA). All experimental treatments were performed three times independently. Group-wise comparisons were subsequently assessed using Tukey’s HSD procedure. Data distribution normality was verified using the Shapiro–Wilk approach for each group (*n* = 5), yielding *p* values between 0.09 and 0.95. In addition, hierarchical cluster analyses based on data normalization were implemented in OriginPro 2022 (OriginLab Corp., Northampton, MA, USA).

## 3. Results

The effect of ultrasound power during the self-assembly process on the multi-scale supramolecular structure and in vitro anti-digestibility of nano-scale EFA ternary assemblies was comprehensively studied.

### 3.1. Self-Assembly Properties Analysis

**Figure 1:**
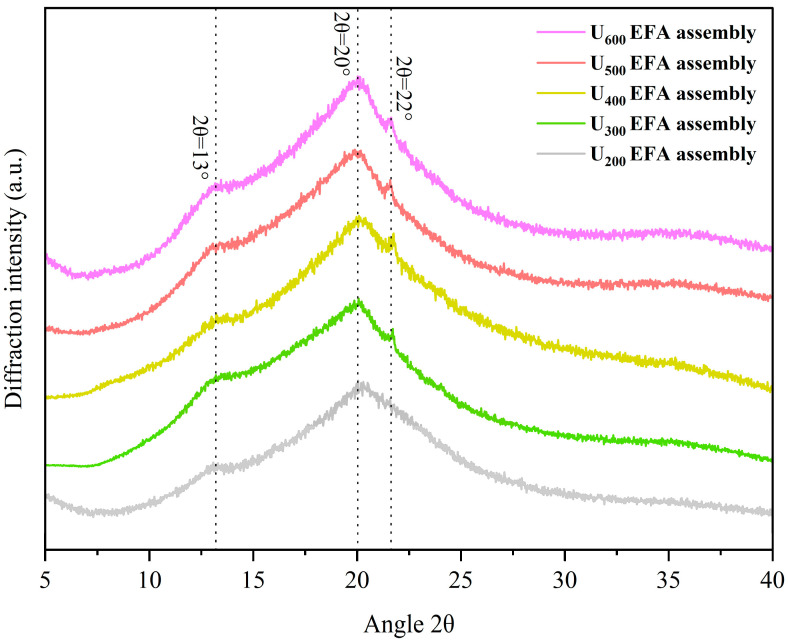
XRD diffraction patterns of nano-scale EFA assemblies prepared under varying ultrasonic power levels (200–600 W). The crystalline peaks were indicated by dashed lines.

**Figure 2:**
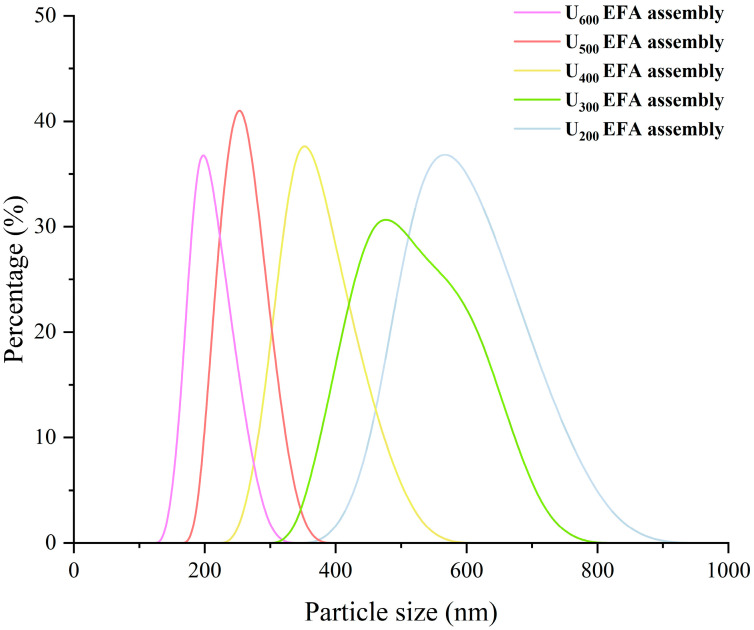
Intensity-weighted hydrodynamic particle size distributions of the nano-scale EFA ternary assemblies.

Qualitative analysis of the self-assembly properties of EFA–LA–βLG assemblies was performed using 2D–COS FTIR and XRD peak analysis. 2D FTIR refers to two-dimensional correlation analysis performed on FTIR spectra using asynchronous 2D-COS. The asynchronous spectra were constructed from the intensity differences of FTIR bands between samples, aiming to highlight non-synchronous variations in characteristic vibrational modes arising from structural differences. Therefore, combined analysis of 2D-COS FTIR and 1D FTIR could better verify the formation of EFA ternary assemblies. As shown in Figures S1 and S2, both 2D-COS FTIR and 1D FTIR showed characteristic cross-peaks around 1540 and 2846 cm^−1^. The band at approximately 1540 cm^−1^ was assigned to the amide II vibration of βLG, mainly arising from N–H bending coupled with C–N stretching, while the band at approximately 2846 cm^−1^ corresponded to the symmetric stretching vibration of methylene groups in LA, reflecting the involvement of aliphatic hydrophobic chains [39,40]. Such correlated responses indicated strong noncovalent interactions, particularly hydrophobic interactions, between βLG and LA within the EFA ternary assemblies. And with increasing ultrasonic power, the asynchronous peaks of the corresponding functional groups increased (Figure S1), indicating that the number of assembly sites in EFA assemblies increased accordingly. In addition, Figure 1 shows that U_200–600_ EFA ternary assemblies display V-type ternary assemblies (2θ ≈ 13°, 20°, and 22°), according to Ji et al. [40]. However, although the U_200_ EFA ternary assembly also displayed a typical V-type crystalline structure, only an extremely weak diffraction signal was observed at 2θ ≈ 22°. This phenomenon might be attributed to the relatively small helical cavity volume and weaker electrostatic potential during self-assembly, which limited the incorporation of lauric acid alkyl groups within the internal cavity. Moreover, as displayed in Figure 2, the particle size distribution of all EFA assemblies was within the range of 100 to 800 nm, suggesting that all samples consist of micro-nano particles. Overall, 2D–COS FITR, XRD peak analysis, and particle size distribution revealed that the EFA side chain could interact with the hydroxyl, amide, and polar side of βLG and LA through noncovalent interaction and further form the nano-scale EFA–LA–βLG assemblies.

**Figure 3:**
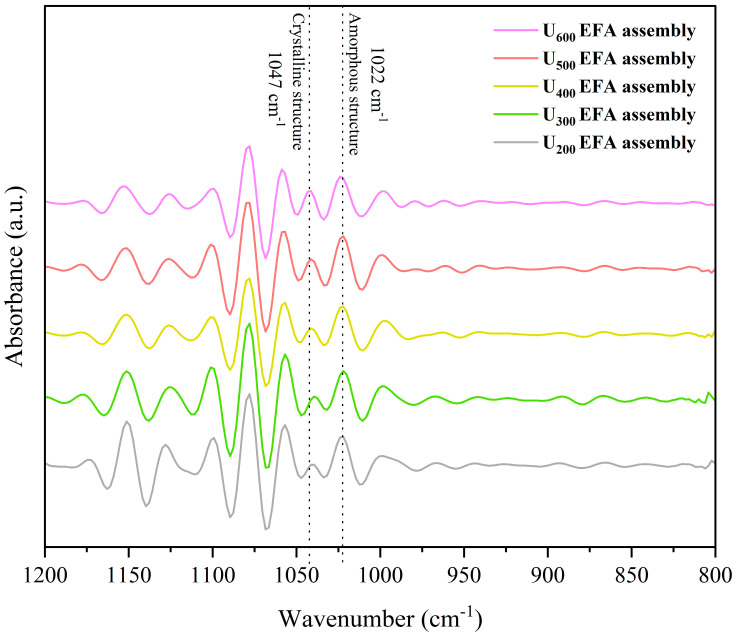
Fourier self-deconvoluted spectra obtained from the FTIR 1200–800 cm^−1^ region of nanoscale EFA ternary assemblies prepared under ultrasonic power levels of 200, 300, 400, 500, and 600 W. The crystalline region-related ordered structures and amorphous structure were indicated.

The quantitative analysis of the EFA assemblies’ self-assembly properties were evaluated by SI, *R_c_*, short-range order, and particle size. Among them, the short-range order was evaluated based on the 1047/1022 cm^−1^ ratio in Figure 3. The band at 1047 cm^−1^ was assigned to the ordered structures, while the band at 1022 cm^−1^ corresponded to the amorphous structures. The outcomes exhibited an increase in SI, *R_c_*, and short-range order of EFA assemblies as the ultrasound power increased (Figure 1 and Figure 3). This was accompanied by a decrease in micro-nano particle size distribution (597.84 to 189.20 nm) (Figure 2 and Table 1). These results indicated that, with the increase in ultrasound power, the dominant mechanism during the high-temperature gelatinization stage of EFA self-assembly transitioned from simple thermal effects to a combined dominance of ultrasonic micro-jet and thermal effects. The ultrasonic cavitation effect intensified, leading to a more effective disruption of the hydrogen bonds between raw EFA chains. This increased molecular thermal motion and led to more dispersed and extended the side chain [29]. As a result, the number of assembly sites in EFA assemblies increased, leading to a rise in SI with increasing ultrasonic power. This induced the formation of a larger number of ordered and compact microcrystalline cross-linked networks [14], which accounted for the increase in *R_c_* and short-range order, as well as the decrease in particle size distribution. Moreover, the SI, short-range order, and particle size values of nano-scale EFA ternary assemblies were broadly similar to those reported previously for maize amylopectin ternary assemblies (19.60% to 53.06%, 252.57–582.63 nm, and 0.21–0.61) [14]. The *R_c_* in the present investigation fell within the published range for various starch assemblies (10.9–33.2%) [23].

Moreover, this investigation further examined the supramolecular structure, thermal behavior, viscosity characteristics, and digestive properties to elucidate the effects of ultrasonic power during the self-assembly process on multi-scale structural organization and digestibility.

### 3.2. Supramolecular Structure Analysis

#### 3.2.1. Chain Length Distribution and Self-Assembly Sites Analysis

**Figure 4:**
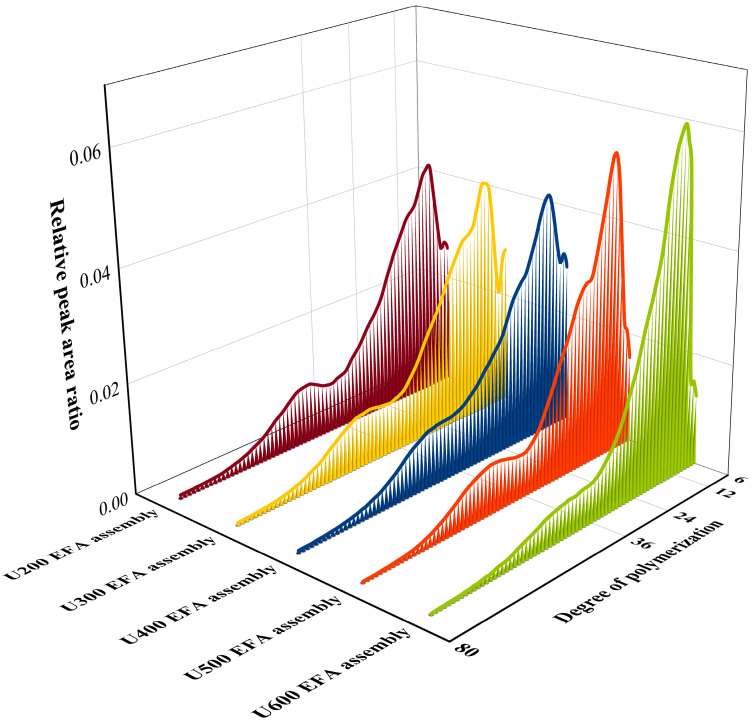
Debranched amylopectin chain length distributions of EFA assemblies determined via HPAEC–PAD.

The fine structure was analyzed based on the chain length distribution, including short side chains (A chains, DP = 6–12), medium–short side chains (B1 chains, DP = 13–24), medium–long side chains (B2 chains, DP = 25–36), and long side chains (C chains, DP > 25–36) [17]. The changes in the chain length distribution of starch assemblies were further assessed to infer the distribution of self-assembly sites along the side chains [24]. Figure 4 and Table 2 showed that, with increasing ultrasonic power, the B2 and C chains of nanoscale EFA assemblies decreased significantly, leading to a reduction in CL. These findings indicated that higher ultrasound power during ternary self-assembly substantially increased the number of active hydroxyl groups on EFA long side chains (B2 and C chains), exposing more self-assembly sites [18,41]. Therefore, the B2 and C chains of EFA can serve as self-assembly sites to form nano-scale EFA ternary assemblies. However, the relative contents of A and B1 chains displayed a disordered change after high-power ultrasound treatment (Figure 4 and Table 2). This phenomenon suggested that short chains (A and B1 chains) could not serve as assembly sites, as the resulting cavity size was too small to accommodate LA and βLG subunits [26]. Additionally, the A and C chains of nano-scale EFA ternary assemblies differed from those reported previously for maize starch ternary assemblies (6.78–20.63% and 21.43–31.60%) [42]. This difference could be attributed to variations in the amylopectin branching degree between EFA and maize starch, which were caused by different types of branching enzymes (BE IIb and IIa).

#### 3.2.2. Semicrystalline Lamellar Structure

**Figure 5:**
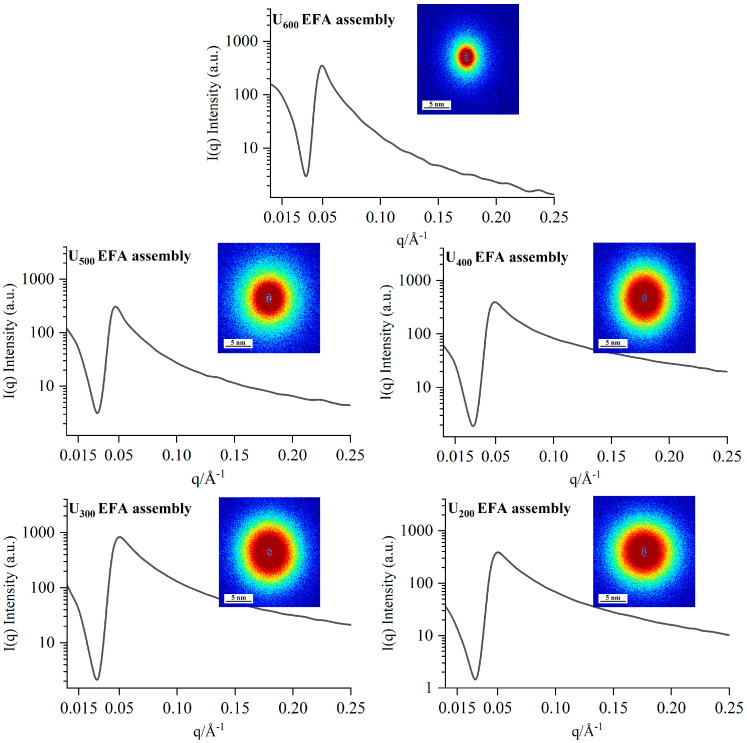
The 2D SAXS scattering patterns and 1D correlation function curves of the EFA ternary assemblies. These were presented to illustrate the semicrystalline lamellar structural properties of the samples.

The semicrystalline lamellar structure of EFA ternary assemblies can be analyzed by SAXS. Initially, the Debye ring of the 2D SAXS pattern was used to roughly visualize the semicrystalline structure. Figure 5 shows that all samples displayed near-circular scattering rings with high irregularity and inhomogeneity, which suggested that the nano-scale EFA ternary assemblies possessed a non-periodic structure [38]. Furthermore, a prominent scattering peak was observed at q ≈ 0.05 Å^−1^, which corresponds to an internal periodic nanostructure with a d-spacing of approximately 13 nm (Figure 5) [12], indicating that the EFA ternary assemblies possess nano-scale structures.

Furthermore, the 1D correlation function curves calculated from the Debye rings enabled a more precise analysis of the semicrystalline lamellar structure (Figure 5). As the ultrasound power increased, *d*, *d_a_*, *d_c_*, *D_m_*, *ξ*, and *ξ_c_* values of the EFA assemblies obtained from the 1D correlation function curves showed a decreasing trend (Figure 5 and Table 3). These outcomes suggested that high-power ultrasound facilitated an increase in electron cloud density and a reduction in hydrogen bond range within the fine structure of the EFA assemblies [12], leading to an increase in SI and a subsequent decrease in the semicrystalline lamellar thickness of the EFA samples. This reorganization into more favorable ordered assemblies resulted in higher short-range order and lower *D_m_* with increasing ultrasonic power. Published reports found slightly different results for *d*, *d_a_*, *d_c_*, *D_m_*, *ξ,* and *ξ_c_* of waxy maize starch ternary assemblies (4.81–3.41 nm, 2.35–1.77 nm, 2.32–1.57 nm, 1.91–1.36, 4.06–6.18 Å and 3.46–4.58 Å) [14]. These variations can be attributed to differences in the sensitivity and operational parameters of 2D-SAXS measurements across different instruments, as well as to differences in the number and distribution of chain self-assembly sites on the free side chains between EFA and waxy maize starch.

#### 3.2.3. Molecular Configuration and Conformation

**Figure 6:**
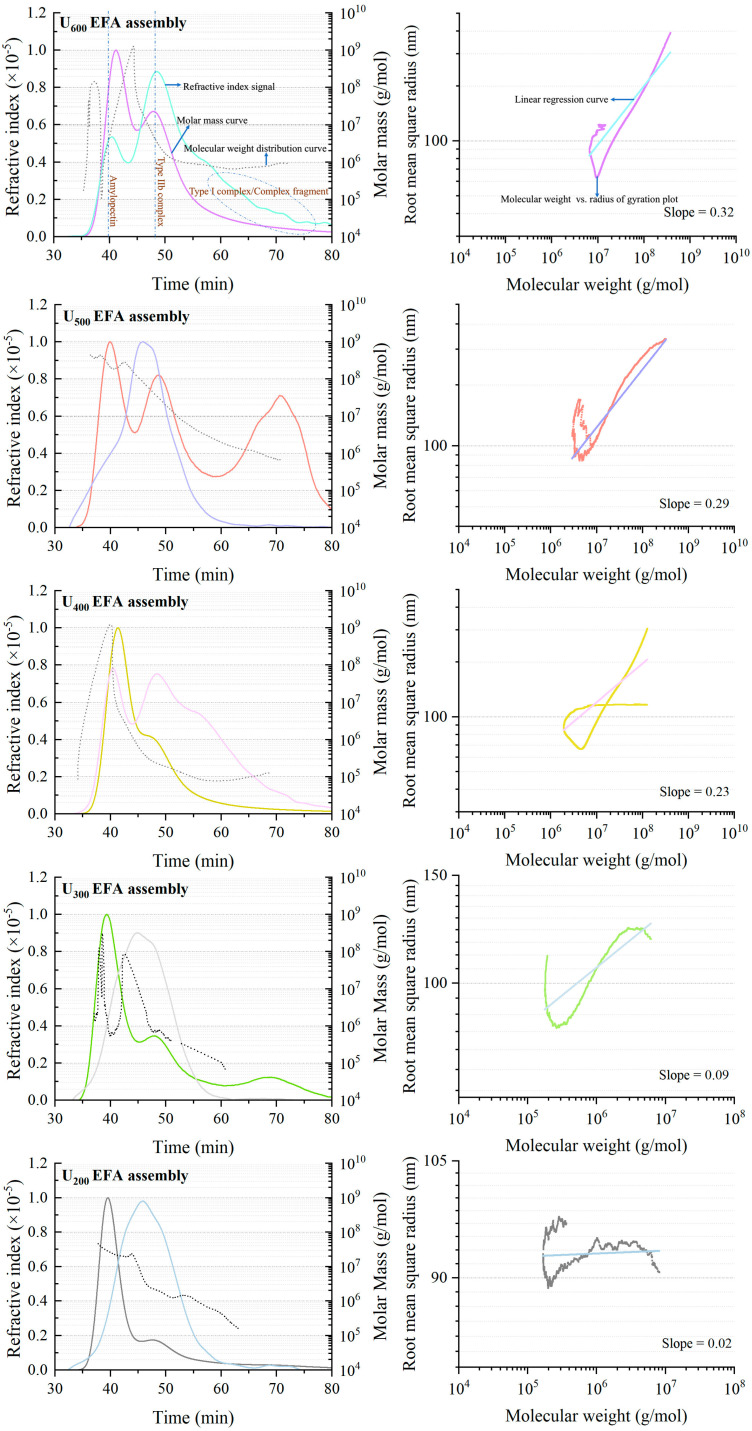
Molecular weight distribution plot (**left**) and molecular configuration plot (**right**) for nanoscale EFA ternary assemblies prepared under varying ultrasonic power. The molecular weight distribution consisted of the RI signal, the molar mass curve, and the Mw distribution curve. The molecular configuration (**right**) was constructed by logarithmically transforming the Mw and Rg data. The molecular weight values corresponding to different components were indicated in the molecular weight distribution plot.

The supramolecular structure of EFA assemblies under different high-power ultrasound conditions was evaluated from molecular weight distribution and molecular configuration. As displayed in Figure 6, the radius of gyration (*R_g_*) vs. *M_w_* fitting curve indicated that the supramolecular conformations transformed from a loosely organized, irregular coil-like state (molecular conformation index, *ν* = 0.02–0.09) toward a more compact, near-spherical structure with increasing ultrasonic power (*ν* = 0.23–0.32) [13]. The looped curves of EFA assemblies in molecular configuration curves could be attributed to the high polydispersity and non-uniform molecular weight distribution (Figure 6). The RI curves of the U_600_ EFA assembly, U_500_ EFA assembly, U_400_ EFA assembly, and U_300_ EFA assembly show two or three molecular weight peaks, suggesting the existence of EFA (≈10^5^–10^8^ Da), type IIb EFA assemblies (≈10^7^–10^9^ Da), and type I EFA assemblies (≈10^5^–10^7^ Da) (Figure 6) [24]. In contrast, the RI curve of the U_200_ EFA assembly showed only one broad peak (molecular weight 10^5^ to 10^9^ Da). This phenomenon could be due to the low SI of the U_200_ EFA assembly and the wide molecular weight distribution of amylopectin, resulting from the presence of numerous fragments with different chain lengths.

Table 4 provides a quantitative summary of the supramolecular parameters extracted from Figure 6. Among them, the number-average molecular mass (*M_n_*), *M_w_*, *R_g_*, and molecular density (*ρ*) increased as the ultrasound power rose. These parameters collectively demonstrated that increasing the ultrasound power promoted higher molecular weight, increased compactness, and enhanced the configurational order of the EFA ternary assemblies. Importantly, the evolution of these supramolecular parameters was consistent with the structural trends observed in other analyses, including increased SI, short-range order, and *R_c_* [10]. Moreover, significantly lower *v* values were observed for the U_200_ and U_300_ EFA assemblies compared to the others (Figure 6). This phenomenon could be attributed to the fact that, compared to the high-power ultrasound-treated assemblies, the samples prepared under lower power exhibited a markedly lower number and sparser distribution of assembly sites within the amylopectin. Consequently, the SI of U_200_ and U_300_ EFA assemblies remained lower, leading to a more irregular distribution of microcrystalline units within the supramolecular structures. This structural disorder likely induced larger molecular torsion angles in the lower-power samples than in the high-power samples, resulting in its more homogeneous yet disordered and loosely packed molecular configuration. In contrast, the PI values of all samples exhibited disordered patterns (Figure 6 and Table 4). This could be attributed to disorder-induced changes in the distribution of A and B1 chains with increasing ultrasound power, which led to the formation of unstable type I EFA assemblies and caused a random arrangement of semicrystalline lamellae. The *M_w_* and PI of the nano-scale EFA ternary assemblies were comparable to previous results for chempedak starch ternary assemblies (3.68–4.77 × 10^7^ Da and 1.98–2.13) [24].

#### 3.2.4. The Microstructure and Nano-Surface Texture

**Figure 7:**
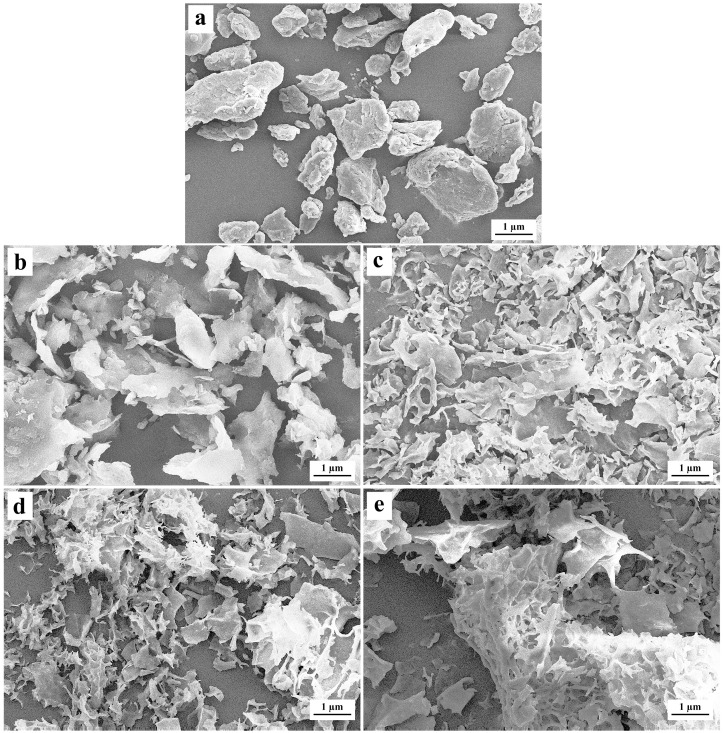
SEM micrographs of nanoscale EFA assemblies prepared under varying ultrasonic power levels, observed at 30,000×. The ultrasonic power increased from left to right: (**a**–**e**) = 200 W, 300 W, 400 W, 500 W, and 600 W.

The microstructure is displayed in the SEM images in Figure 7. All the nano-scale EFA ternary assemblies exhibited irregular particle morphology with uneven size, sponge-like features, and emulsion-like protrusions. This phenomenon could be attributed to the fact that the starch self-assembly required complete gelatinization of starch granules at the initial stage [10]. During this process, the starch granules underwent extensive swelling, lost their native granular morphology, and experienced melting of the crystalline regions, leading to the complete dissociation of amylopectin chains [12]. After self-assembly, these dissociated chains were thoroughly rearranged, ultimately forming randomly particulate morphologies. Consistently, the multimodal molecular weight distribution and 2D-SAXS results indicating “non-periodic structures” strongly support this interpretation. Moreover, with increasing ultrasound power during self-assembly, a reduced number of pores and protrusions, as well as decreased sponge-like features, surface fractal dimensions, and surface roughness were observed in the EFA assemblies’ SEM images (Figure 7). Similar nano-scale near-surface features can be observed in the Supplementary S2 AFM images (Figure S3 and Table S2), which provide supportive morphological reference information.

### 3.3. DSC Analysis

**Figure 8:**
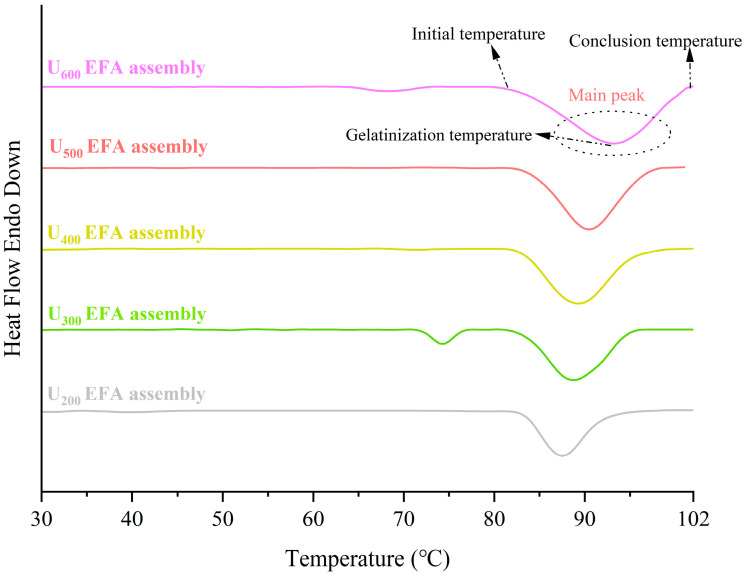
DSC curves of nano-scale EFA assemblies. The main peaks, including initial to conclusion temperature, are indicated.

The gelatinization characteristic parameters of nano-scale EFA ternary assemblies were calculated through DSC curves, which were used to reflect the melting characteristics of the crystalline structure. As shown in Figure 8 and Table 5, two crystalline melting peaks appeared for U_300_ EFA assemblies and U_600_ EFA assemblies, presenting the type I assemblies or starch paste and the type IIb assemblies’ peaks [39]. Previous research reported that the melting temperature ranges for Type I and Type IIb assemblies of rice starch fell within 70.17–92.17 °C and 84.73–114.32 °C [3], respectively, which was consistent with these results. However, U_200_ EFA assemblies, U_400_ EFA assemblies, and U_500_ EFA assemblies only showed one DSC curve peak. This might be attributed to the relatively low molecular weight of type I assemblies and starch paste fragments, which did not reach the sensitivity limit of the instrument. In the main peak, the *T_o_*, *T_p_*, *T_c_*, *R*, and Δ*H_g_* values of the nano-scale EFA ternary assemblies increased with the increasing ultrasound power (Figure 8 and Table 5). These results indicated that the resistance of the crystalline structure of EFA assemblies to melting increased significantly with increasing ultrasonic power. The shorter hydrogen bond lengths, strengthened intramolecular hydrophobic interactions, improved homogeneity of double helices, and the increased number and ordered arrangement of long double helices in EFA assemblies with increasing ultrasonic power can explain this phenomenon [25]. The resulting strengthened intramolecular force increased the thermal stability of starch particles. The *T_o_*, *T_p_*, *T_c_*, *R*, and Δ*H_g_* were broadly similar to the reported values of chempedak seed amylopectin ternary assemblies (80.05 °C, 92.71 °C, 98.85 °C, 14.41 °C, and 14.41 J g^−1^) [24].

### 3.4. Viscous Properties

**Figure 9:**
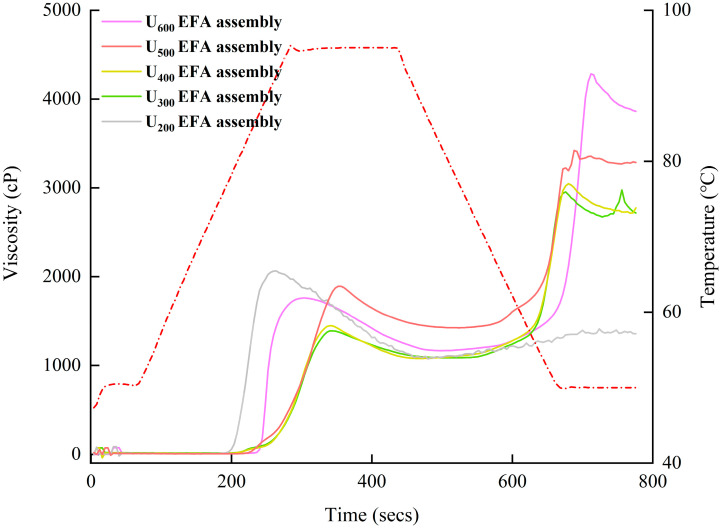
Viscous property curves of nano-scale EFA assemblies. The red dashed line represents the recorded temperature–time profile, and the colored solid lines represent the viscosity curves obtained during the RVA analysis.

The viscosity curves shown in Figure 9 were generated by the RVA built-in program and subsequently replotted using Origin 2022b software. As shown in Figure 9 and Table 6, nano-scale EFA assemblies prepared under higher ultrasonic power exhibited significantly higher viscosity related parameters, including BDV, FV, SBV, and PT, compared to samples prepared at lower power levels. These phenomena could be explained by that the water-holding capacity of the EFA assemblies prepared under higher ultrasonic power was weakened during gelatinization, retarding molecular swelling and reducing the melting ability of the crystalline structures [23,24]. As a result, this facilitated the rapid reconstruction of intermolecular hydrogen bonds during retrogradation, enhanced the re-association capability of the fine structure, and increased molecular chain rigidity, strengthening the cross-linking degree of the molecular gel network [41]. Additionally, the PV and TV of the nano-scale EFA assemblies showed a chaotic trend with the change in ultrasound power (Figure 9 and Table 6). The above outcomes may be attributed to the disordered PI, which resulted in the instability of the EFA assemblies under thermal and mechanical shear stress. Previous researchers found similar values of PV (1123.0–3789.0 cP), TV (684.0–1387.0 cP), BDV (360.0–2402.5 cP), FV (1464.0–2499.0 cP), and SBV (188.0–1412.0 cP) for wheat starch assemblies [26].

### 3.5. In Vitro Digestible Characteristics

#### 3.5.1. Digestive Fraction

The in vitro digestible characteristics mainly included RDS, SDS, and RS, digestive kinetics, and EGI analysis. As shown in Table 7, all the nano-scale EFA assemblies were classified as high RS-V content starches (RS > 15%) [4]. Along with the increase in ultrasound power on the nano-scale EFA assemblies, many RDS structures (61.48–15.64%) were transformed into SDS and RS-V structures (16.86–24.19% and 22.66–60.17%) (Table 7). This indicated that high-power ultrasound treatment not only significantly enhanced the anti-digestibility of EFA assemblies but also simultaneously sustained the release of the three major nutrients. Based on previous report [2,7,15], RDS, SDS, and RS-V structures corresponded to the amorphous regions, partial-order crystalline regions, and perfect spherulitic domains, respectively. Therefore, the transformation from RDS into SDS and RS-V structures during high-power ultrasound could result from the higher *R_c_*, generating pronounced hydrophobic effects that reduced both the contact area and the number of glycosidic bond cleavage sites. The SDS and RS-V of the nano-scale EFA assemblies in the present research were higher than those of the corn, maize, cassava, potato, and rice starch assemblies (5.74–15.51% and 12.90–24.08%, respectively) [15,27]. This difference might be attributed to the differences in SI, hydrophobic interactions, *ρ,* and nano-surface texture between the EFA assemblies and the reported staple crop starch assemblies.

#### 3.5.2. Investigation of In Vitro Digestive Kinetics and EGI

**Figure 10:**
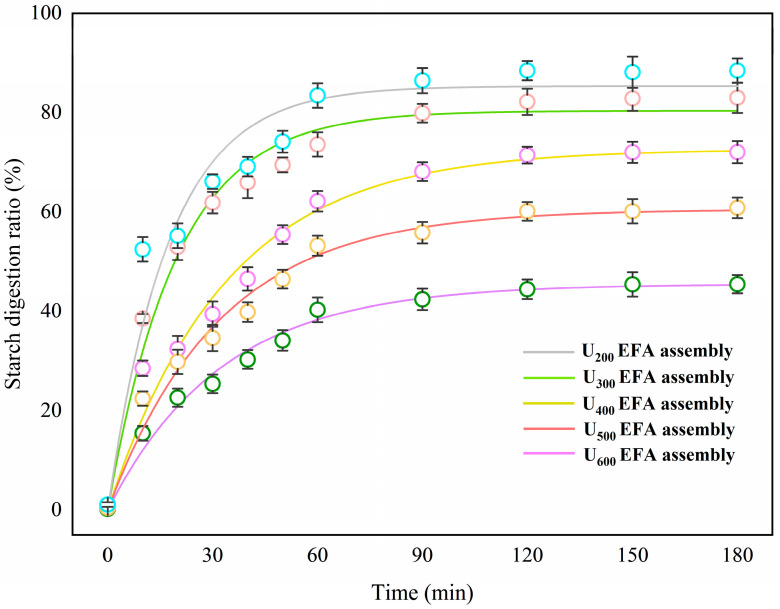
In vitro intestinal digestion profiles of EFA ternary assemblies prepared under different ultrasound power levels, showing digestive ratios as a function of time.

**Figure 11:**
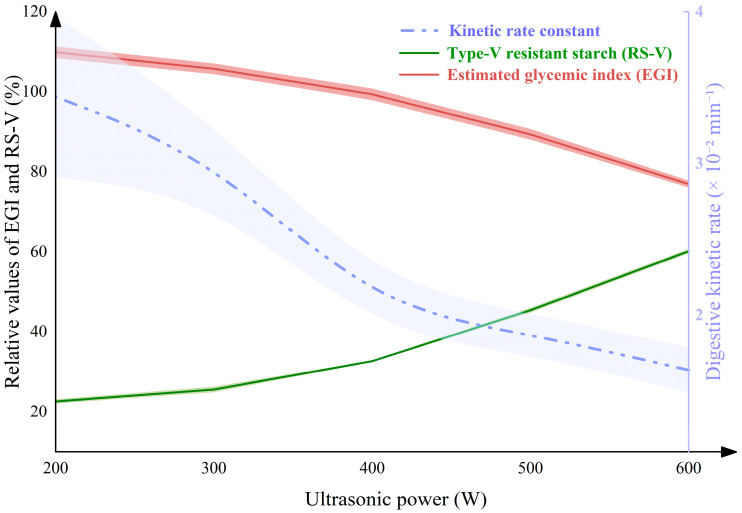
The relationship between RS-V, *k* and EGI of EFA ternary assemblies prepared under high-power ultrasound. The colored regions represent shaded error bands.

Since digestive fractions could significantly influence the digestive process, the digestive kinetics of the nano-scale EFA ternary assemblies was further investigated. As displayed in Figure 10 and Table 7, increasing ultrasound power during self-assembly decreased the *C*_∞_ and *k* (3.44–1.64 × 10^−2^ min^−1^) of nano-scale EFA ternary assemblies, leading to the decreases in HI and EGI (109.88–76.98). These outcomes are in agreement with the finding that the glycemic release of nano-scale EFA assemblies became more stable and reduced the risk of developing diabetes after high-power ultrasound [39]. Overall, increasing ultrasonic power enhanced RS-V formation and digestive resistance, thereby reducing the glycemic response and slowing the digestion rate (Figure 11). These outcomes could be explained by the fact that, as ultrasonic power increased, SI, tightly packed trans-lamellar chains, and organized V-type helical content increased [37]. This led to a decrease in the sizes of internal and external hydrolysis channels [36]. As a result, the enzymatic hydrolysis pattern might have shifted from an inside-out mode to an outside-in mode. The lower broad *k* values of the nano-scale EFA ternary assemblies were lower than those of the published breadfruit and rice starch ternary assemblies (1.68–8.06 × 10^−2^ min^−1^) [12,13]. Compared to the reference samples, the more compact molecular cross-linking network of EFA ternary assemblies enhanced hydrophobic interactions, hindering the rapid penetration of digestive enzymes and providing a plausible explanation for these differences.

However, the in vitro digestive model employed in this study was designed to simulate intestinal starch digestion and focused on enzyme-mediated hydrolysis under controlled intestinal conditions. Accordingly, the model simplified the intestinal environment by applying fixed enzyme concentrations and did not fully account for dynamic intestinal mixing. Therefore, the EGI values derived from this model should be interpreted as indicators of relative intestinal digestion behavior rather than direct physiological glycemic responses. Future work will focus on refining intestinal digestion conditions and further validating the observed digestion trends using more physiologically relevant intestinal models.

### 3.6. Investigation of the Influence of Ultrasound Power on Anti-Digestibility Mechanisms

#### 3.6.1. 2D PCA

**Figure 12:**
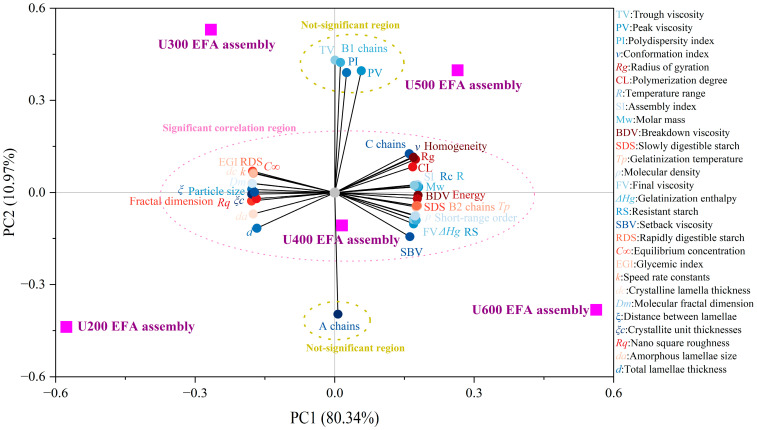
2D PCA models were constructed for EFA ternary assemblies using a correlation-based approach. Purple squares denoted samples treated with various ultrasonic powers, distributed based on their PCA scores. Significantly correlated regions and non-significant regions of digestibility, physicochemical properties and multi-scale structural features were highlighted in the figure.

2D PCA and hierarchical cluster analyses were performed to reveal the correlations between digestibility and the structure of nano-scale EFA ternary assemblies prepared under various ultrasound powers. As seen in Figure 12, U_600_ EFA assembly, U_500_ EFA assembly, U_400_ EFA assembly, U_300_ EFA assembly, and U_200_ EFA assembly were widely distributed in the 2D PCA space, indicating that high-power ultrasound could significantly influence the structure and digestibility properties of nano-scale EFA ternary assemblies [13]. The cumulative contribution of PC1, PC2, and PC3 exceeded 99%, indicating that the 2D PCA model effectively captured the overall structure of the dataset (Figure 12) [14]. As displayed in 2D PCA and its corresponding correlation plot (Figure 12 and Figure S4), a significant positive correlation was exhibited for RS-V, SDS, SI, *R_c_*, short-range order, *M_w_*, *ρ*, *ν*, *R_g_*, *T_p_*, Δ*H_g_*, *R*, SBV, BDV, FV homogeneity, and energy (angle < 45°, r > 0.82, *p* < 0.05). Similarly, *d*, *d_a_*, *d_c_*, *ξ*, *ξ_c_*, particle size, fractal dimension, *R_q_*, *D_m_*, B2 chains, C chains, CL, RDS, *C*_∞_, *k*, and EGI also exhibited a significant positive correlation (angle < 45°, r > 0.82, *p* < 0.05). Moreover, variables in region I including RS-V, SDS, SI, *R_c_*, short-range order, *M_w_*, *ρ*, *ν*, *R_g_*, *T_p_*, Δ*H_g_*, *R*, SBV, BDV, FV, homogeneity, and energy showed significant negative correlations with those in region II comprising *d*, *d_a_*, *d_c_*, *ξ*, *ξ_c_*, particle size, fractal dimension, *R_q_*, *D_m_*, B2 chains, C chains, CL, RDS, *C_∞_*, *k*, and EGI (angle > 45°, r < −0.82, *p* < 0.05). Additionally, TV, PV, PI, A chains, and B1 chains in other regions indicated the weak correlations with other variables (Figure 12). Overall, the 2D PCA results suggested that high ultrasonic power could optimize self-assembly properties, render the molecular configuration and conformation more compact and ordered, and improve gelatinization behavior and gel network formation, thereby decreasing digestibility with increasing ultrasonic power. Wang et al. [9] also reported that SI exhibited a significant positive correlation with both the number and distribution area of assembly sites. Chao et al. [10] confirmed that starch assemblies with higher SI possessed a greater number of hydrogen bonds and stronger noncovalent interactions. In addition, Li et al. [14] reported that starch assemblies with higher SI exhibited lower digestion kinetics.

#### 3.6.2. Investigation of Hierarchical Cluster

**Figure 13:**
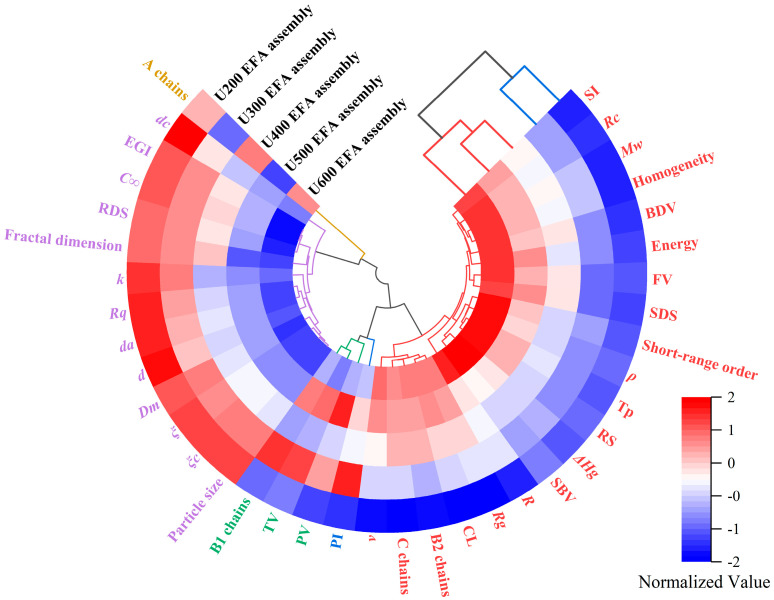
The circular hierarchical cluster dendrogram illustrated the interrelationships among the digestibility, physicochemical properties, and structural characteristics of the nanoscale EFA assemblies prepared under varying ultrasonic power levels. The circular dendrogram located at the center represents the interrelationships among the characteristic parameters, while the square dendrogram at the periphery represents the clustering relationships among the samples. M Moreover, the abbreviations shown in the figure are defined as follows: TV, trough viscosity; PV, peak viscosity; PI, polydispersity index; *ν*, conformation index; *R_g_*, radius of gyration; *R_c_*, residual crystallinity; CL, polymerization degree; *R*, temperature range; SI, assembly index; *M_w_*, molar mass; BDV, breakdown viscosity; SDS, slowly digestible starch; *T_p_*, gelatinization temperature; *ρ*, molecular density; FV, final viscosity; Δ*H_g_*, gelatinization enthalpy; RS, resistant starch; SBV, setback viscosity; RDS, rapidly digestible starch; *C*_∞_, equilibrium concentration; EGI, estimated glycemic index; *k*, speed rate constants; *d_c_*, crystalline lamella thickness; *D_m_*, molecular fractal dimension; *ξ*, distance between lamellae; *ξ_c_*, crystallite unit thicknesses; *R_q_*, nano square roughness; *d_a_*, amorphous lamellae size; *d*, semicrystalline lamellae thickness.

The circular hierarchical cluster dendrogram demonstrates the similarities among different nano-scale EFA ternary assemblies and the correlations among various parameters [38]. As shown in Figure 13, the U_600_ EFA assembly exists in an independent cluster, while the U_500_ EFA assembly and U_400_ EFA assembly, as well as the U_300_ EFA assembly and U_200_ EFA assembly, are grouped in the same cluster (Figure 13). These findings demonstrate that, with increasing ultrasound power, the effects on multi-scale assembly structural features, physicochemical characteristics, and digestive properties become increasingly pronounced. This result was similar to the wide distribution of samples observed in the PCA. Cluster branch I included the A chains. Cluster branch II included *d*, *d_a_*, *d_c_*, *ξ*, *ξ_c_*, particle size, fractal dimension, *R_q_*, *D_m_*, B2 chains, C chains, CL, RDS, *C_∞_*, *k*, and EGI. B1 chains, TV, and PV comprised branch III. Branch IV contained PI. Cluster branch V consisted of RS-V, SDS, SI, *R_c_*, short-range order, *M_w_*, *ρ*, *ν*, R_g_, *T_p_*, Δ*H_g_*, *R*, SBV, BDV, FV, homogeneity, energy, B2 chains, C chains, and CL (Figure 13). Based on this, cluster branches I, III, and IV appeared isolated, suggesting that A chains, B1 chains, TV, PV, and PI exhibited no significant interactions with the other variables [24]. The result was also similar to those observed in the PCA (Figure 12). Cluster branches II and V contained most of the research parameters, with variables within each cluster displaying strong mutual correlations. This clustering pattern confirmed the marked relationship among the multi-scale assembly structural features, physicochemical characteristics, and digestive properties of the EFA ternary assemblies prepared under high ultrasound power. However, cluster branch V, comprising CL, B2 chains, and C chains, showed slight differences compared with the PCA (Figure 12). This is because a circular hierarchical cluster, based on the Euclidean method, directly measures the overall normalized similarity among samples and can capture nonlinear relationships, whereas PCA focuses on the hierarchical structure of samples and the strength of the correlations [38].

**Figure 14:**
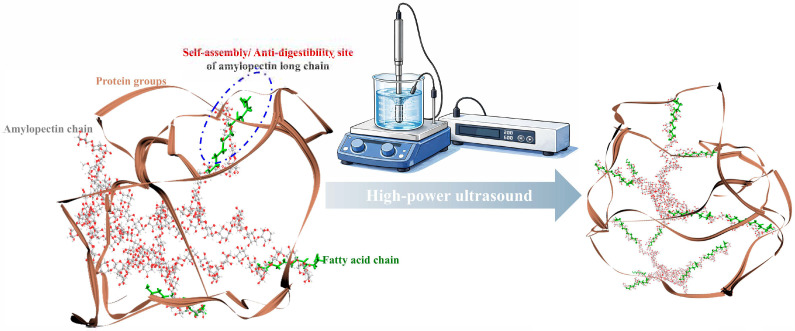
Schematic representation of the proposed ultrasound-mediated self-assembly mechanism of EFA ternary assemblies based on the experimental results of multi-scale supramolecular structure. Before high-power ultrasonic treatment, the initial structure possessed a low side-chain density and a disordered, coil-like configuration. These features reduced steric hindrance and exposed more enzymatic hydrolysis sites within a loose conformational state. After high-power ultrasonic treatment, a large number of assembly sites was generated within EFA, which strengthened intramolecular non-covalent interactions. The side-chain density of the EFA ternary assemblies increased markedly, driving the formation of a more ordered and compact configuration. Consequently, the final assemblies exhibited a highly dense and organized conformation.

Based on a combination of the 2D PCA and the clustering heatmap, the influence of ultrasound power during the self-assembly process on the multi-scale supramolecular structure and anti-digestibility was comprehensively inferred. Figure 14 illustrates that the increase in ultrasound power during self-assembly enhanced the distribution and number of assembly sites within EFA B2 and C chains. [24]. Meanwhile, electron cloud density was increased, strengthening intramolecular noncovalent interactions [35,41]. As a result, the increasing SI was observed. This encapsulated the enzymatic cleavage sites. [18,40]. Subsequently, a decrease in the helical cavity volume and the shortened hydrogen bonding distances was inferred [23]. This could allow the abundant formation of order arrangement long helical structures, leading to the increased number of ordered V-type crystalline structures [40]. Therefore, lower values for *d*, *d_a_*, *D_m_*, *ξ*, and *ξ_c_* were observed with the increase in ultrasound power [14]. The increased semicrystalline lamellar density and the extensive formation of spherulitic inferred from the above results significantly increased the *R_c_* and short-range order. Therefore, increasing the ultrasound power promoted the extensive transformation of RDS structures into SDS and RS-V structures. These results suggested that the density of the microcrystalline cross-linking network increased with increasing ultrasound power, leading to increases in *M_w_*, *R_g_*, and *ρ* and implying increases in the BDV, FV, and SBV of EFA assemblies [10,24]. This caused an ordered transition of molecular configuration and conformation. Overall, the distribution of enzymatic hydrolysis channels on the particle surface was reduced, which markedly decreased *k* and *C_∞_*. This led to a substantial decrease in the EGI by increasing the ultrasonic power applied to the EFA assemblies. Additionally, compared to white waxy maize, waxy potato, waxy wheat, and waxy rice starch ternary assemblies prepared under the high-power ultrasound, higher values of RS-V and SDS were shown for the nano-scale EFA ternary assemblies (Table S3). This led to the lower *k*, *C_∞_*, HI, and EGI of the nano-scale EFA ternary assemblies than those of white waxy maize, waxy potato, waxy wheat, and waxy rice starch ternary assemblies. These results indicate that *Euryale ferox*, as a non-traditional resource, contains amylopectin with substantial potential for assembly and modification compared with that from staple crops.

## 4. Conclusions

In order to investigate the influence of high-power ultrasound on the supramolecular structure and anti-digestibility of amylopectin assemblies, EFA was used as the starting material to self-assemble with βLG–LA, and nanoscale EFA ternary assemblies were constructed using high-power ultrasound. The multi-scale structure and digestibility of the EFA ternary assemblies were characterized. The results showed that the EFA ternary assemblies prepared under varying ultrasonic power levels all exhibited characteristic self-assembly peaks and V-type crystal structure. Furthermore, enhanced ultrasonic power increased the available assembly sites on the long side chains of the EFA ternary assemblies, thereby raising the self-assembly index (SI). The higher SI facilitated crystalline structure formation and improved the ordering of the semicrystalline structure, leading to higher *M_w_*, *R_g_*, and *ρ*. These structural changes resulted in improved particle morphology, as well as increased *T_p_*, Δ*H_g_*, and viscosity characteristics. Combined PCA and hierarchical clustering further demonstrated that the improved SI, multiscale molecular configuration and conformation, and physicochemical properties of EFA assemblies under increasing ultrasonic power contributed to increased type-V resistant starch (RS-V), along with a decreased digestion rate and estimated glycemic index (EGI). The above analyses verified that high-power ultrasound applied during self-assembly can significantly reduce the nutrient release rate and enhance anti-digestibility properties. Moreover, compared with amylopectin assemblies from staple crops like white waxy maize prepared under high-power ultrasound, EFA assemblies exhibited higher RS-V content and a lower EGI. Although the in vitro digestive model employed in this study effectively characterized the glucose release behavior, it comprised a simplified intestinal environment using fixed enzyme concentrations and did not fully account for dynamic intestinal mixing. Therefore, future work will focus on refining intestinal digestion conditions and further validating the observed digestion trends using more physiologically relevant intestinal models. Overall, this research provides a valuable supplement to current studies on the regulation mechanisms of nano-scale multifunctional anti-digestible starch.

## Figures and Tables

**Table 1:** The self-assembly characteristics of EFA assemblies prepared under various levels of ultrasound power.

Samples	SI (%)	Short-Range Order	*R_c_* (%)	Particle Size (nm)
U_600_ EFA assembly	78.53 ± 1.64 ^a^	0.62 ± 0.04 ^a^	21.76 ± 0.66 ^a^	189.20 ± 2.60 ^e^
U_500_ EFA assembly	70.64 ± 2.25 ^b^	0.48± 0.02 ^b^	19.65 ± 0.84 ^b^	280.14 ± 2.79 ^d^
U_400_ EFA assembly	61.27 ± 2.01 ^c^	0.39 ± 0.03 ^c^	17.92 ± 0.69 ^c^	377.49 ± 6.88 ^c^
U_300_ EFA assembly	50.89 ± 1.55 ^d^	0.35 ± 0.02 ^d^	16.55 ± 0.47 ^cd^	536.25 ± 4.26 ^b^
U_200_ EFA assembly	39.46 ± 1.13 ^e^	0.28 ± 0.02 ^e^	14.70 ± 0.55 ^e^	597.84 ± 3.55 ^a^

SI, self-assembly index; *R_c_*, relative crystallinity. Samples labeled with different superscript letters in the same column differ significantly from one another (*p* < 0.05). Particle size was determined by dynamic light scattering (DLS), and values represent intensity-weighted hydrodynamic diameters.

**Table 2:** Self-assembly characteristics and lamellar parameters of WMS paste and nano-scale ternary assemblies.

Starch Sample	A Chains (DP = 6–12)	B1 Chains (DP = 13–24)	B2 Chains (DP = 25–36)	C Chains (DP ≥ 37)	CL (DP)
U_200_ EFA assembly	23.07 ± 0.10 ^d^	38.07 ± 0.06 ^d^	18.98 ± 0.15 ^a^	19.88 ± 0.08 ^a^	24.31 ± 0.11 ^a^
U_300_ EFA assembly	22.49 ± 0.05 ^e^	39.30 ± 0.15 ^c^	18.73 ± 0.08 ^ab^	19.48 ± 0.10 ^b^	24.10 ± 0.09 ^ab^
U_400_ EFA assembly	24.96 ± 0.08 ^bc^	37.26 ± 0.07 ^e^	18.44 ± 0.21 ^bc^	19.35 ± 0.15 ^c^	23.54 ± 0.14 ^c^
U_500_ EFA assembly	24.75 ± 0.17 ^ab^	41.99 ± 0.22 ^b^	16.60 ± 0.06 ^d^	16.65 ± 0.13 ^d^	22.59 ± 0.16 ^d^
U_600_ EFA assembly	25.04 ± 0.09 ^a^	48.70 ± 0.28 ^a^	14.56 ± 0.04 ^e^	11.70 ± 0.17 ^e^	20.64 ± 0.20 ^e^

DP, degree of polymerization; CL, average degree of polymerization. Samples labeled with different superscript letters in the same column differ significantly from one another (*p* < 0.05).

**Table 3:** SAXS-derived semicrystalline lamellar size of nanoscale EFA ternary assemblies.

Starch Sample	*d* (nm)	*d*_a_ (nm)	*d*_c_ (nm)	*D_m_*	*ξ*	*ξ_c_*
U_600_ EFA assembly	6.69 ± 0.19 ^e^	3.66 ± 0.04 ^e^	3.03 ± 0.19 ^e^	1.71 ± 0.04 ^e^	0.80 ± 0.03 ^de^	1.02 ± 0.04 ^de^
U_500_ EFA assembly	7.59 ± 0.12 ^d^	4.29 ± 0.02 ^d^	3.30 ± 0.12 ^d^	2.11 ± 0.06 ^d^	0.84 ± 0.01 ^d^	1.05 ± 0.02 ^cd^
U_400_ EFA assembly	8.51 ± 0.11 ^c^	4.97 ± 0.07 ^c^	3.54 ± 0.13 ^c^	2.35 ± 0.04 ^c^	0.89 ± 0.02 ^c^	1.09 ± 0.02 ^c^
U_300_ EFA assembly	10.01 ± 0.09 ^b^	5.80 ± 0.06 ^b^	4.21 ± 0.11 ^b^	2.87 ± 0.02 ^b^	0.96 ± 0.02 ^ab^	1.15 ± 0.02 ^ab^
U_200_ EFA assembly	12.57 ± 0.25 ^a^	6.82 ± 0.08 ^a^	5.75 ± 0.26 ^a^	2.98 ± 0.02 ^a^	1.00 ± 0.06 ^a^	1.17 ± 0.08 ^a^

*d*, semicrystalline lamellae thickness; *d_a_*, amorphous lamellae thickness; *d_c_*, crystalline lamella thickness; *D_m_*, mass fractal dimension; ξ, distance between crystalline and amorphous lamellae; *ξ_c_*, crystallite unit thicknesses. Samples labeled with different superscript letters in the same column differ significantly from one another (*p* < 0.05). The size determination was based on a real-space correlation function obtained by converting the 2D SAXS patterns into 1D profiles.

**Table 4:** The molecular configuration parameters of nano-scale EFA ternary assemblies during high-power ultrasound treatment.

Samples	*M_n_* (×10^7^ Da)	*M_w_* (×10^7^ Da)	*R_g_* (nm)	PI	*ν*	*ρ* (g mol^−1^ nm^−3^)
U_600_ EFA assembly	4.04 ± 0.23 ^a^	6.46 ± 0.22 ^a^	160.55 ± 2.88 ^a^	1.60 ± 0.10 ^cd^	0.32 ± 0.02 ^a^	15.61 ± 0.99 ^a^
U_500_ EFA assembly	2.79 ± 0.05 ^b^	5.01 ± 0.16 ^b^	153.55 ± 1.94 ^b^	1.80 ± 0.06 ^b^	0.29 ± 0.03 ^ab^	13.84 ± 0.69 ^b^
U_400_ EFA assembly	2.42 ± 0.06 ^c^	4.14 ± 0.11 ^c^	147.33 ± 1.77 ^c^	1.71 ± 0.06 ^bc^	0.23 ± 0.02 ^c^	12.95 ± 0.58 ^bc^
U_300_ EFA assembly	1.47 ± 0.10 ^d^	3.17 ± 0.09 ^d^	134.90 ± 2.65 ^d^	2.16 ± 0.16 ^a^	0.09 ± 0.02 ^d^	12.37 ± 1.04 ^cd^
U_200_ EFA assembly	0.96 ± 0.07 ^e^	1.25 ± 0.15 ^e^	101.11 ± 1.84 ^e^	1.30 ± 0.18 ^e^	0.02 ± 0.03 ^e^	12.08 ± 1.09 ^de^

*M_n_* and *M_w_*, weight-average and number-average molecular mass; *R_g_*, gyration radius; PI, polydispersity index; *ν*, molecular conformation index; *ρ*, molecular density. Samples labeled with different superscript letters in the same column differ significantly from one another (*p* < 0.05).

**Table 5:** Gelatinization characteristic parameters including *T_o_*, *T_p_*, *T_c_*, *R*, and Δ*H_g_* within the main peak of different nano-scale EFA assemblies.

Samples	*T_o_* (°C)	*T_p_* (°C)	*T_c_* (°C)	*R* (°C)	Δ*H_g_* (J/g)
U_600_ EFA assembly	85.02 ± 0.24 ^a^	91.59 ± 0.81 ^a^	101.52 ± 0.90 ^a^	16.50 ± 0.93 ^a^	18.73 ± 0.63 ^a^
U_500_ EFA assembly	83.07 ± 0.14 ^b^	89.50 ± 0.16 ^b^	97.17 ± 0.12 ^b^	14.10 ± 0.18 ^b^	13.08 ± 0.10 ^b^
U_400_ EFA assembly	81.55 ± 0.34 ^c^	88.14 ± 0.90 ^bc^	95.09 ± 0.49 ^c^	13.54 ± 0.38 ^c^	11.67 ± 0.30 ^c^
U_300_ EFA assembly	80.54 ± 0.25 ^d^	87.09 ± 0.62 ^d^	94.28 ± 0.24 ^d^	13.34 ± 0.25 ^cd^	10.55 ± 0.13 ^d^
U_200_ EFA assembly	82.08 ± 0.19 ^e^	86.51 ± 0.49 ^de^	92.85 ± 0.66 ^e^	10.77 ± 0.69 ^e^	8.56 ± 0.52 ^e^

*T_o_*, initial temperature; *T_p_*, gelatinization temperature; *T_c_*, conclusion temperature; *R*, the gelatinization temperature interval; Δ*H_g_*, gelatinization enthalpy. Samples labeled with different superscript letters in the same column differ significantly from one another (*p* < 0.05).

**Table 6:** The viscous characteristics of the nano-scale EFA assemblies under various levels of ultrasound power.

Samples	PV (cP)	TV (cP)	BDV (cP)	FV (cP)	SBV (cP)	PT (°C)
U_600_ EFA assembly	1760.64 ± 25.17 ^c^	1167.49 ± 30.51 ^e^	593.19 ± 2.95 ^a^	3826.16 ± 20.66 ^a^	2659.80 ± 50.50 ^a^	90.65 ± 0.99 ^a^
U_500_ EFA assembly	1934.84 ± 21.66 ^a^	1452.15 ± 11.66 ^c^	492.18 ± 4.82 ^b^	3432.52 ± 16.50 ^b^	1990.33 ± 13.37 ^b^	87.01 ± 0.51 ^b^
U_400_ EFA assembly	1892.90 ± 10.98 ^b^	1424.19 ± 9.78 ^d^	468.77 ± 10.50 ^c^	3273.33 ± 19.84 ^c^	1849.52 ± 19.65 ^c^	84.32 ± 0.62 ^c^
U_300_ EFA assembly	1447.77 ± 12.49 ^d^	1080.18 ± 4.09 ^ab^	367.62 ± 5.88 ^d^	2774.25 ± 33.15 ^d^	1694.89 ± 18.18 ^d^	83.15 ± 1.00 ^cd^
U_200_ EFA assembly	1390.14 ± 9.08 ^e^	1098.90 ± 15.08 ^a^	303.87 ± 8.91 ^e^	2718.64 ± 16.65 ^de^	1631.70 ± 10.40 ^e^	80.92 ± 0.94 ^e^

PV, peak viscosity; TV, trough viscosity; BDV, breakdown viscosity; FV, final viscosity; SBV, setback viscosity; PT, pasting temperature. Samples labeled with different superscript letters in the same column differ significantly from one another (*p* < 0.05).

**Table 7:** The in vitro digestible characteristics of nano-scale EFA ternary assemblies prepared under various ultrasound power levels.

Samples	RDS (%)	SDS (%)	RS (%)	*C*_∞_ (%)	*k* (×10^−2^ min^−1^)	HI	EGI
U_600_ EFA assembly	15.64 ± 0.20 ^e^	24.19 ± 0.61 ^a^	60.17 ± 0.57 ^a^	45.41 ± 1.07 ^e^	1.64 ± 0.15 ^de^	67.88 ± 1.60 ^e^	76.98 ± 0.88 ^e^
U_500_ EFA assembly	31.81 ± 0.30 ^d^	22.78 ± 0.69 ^b^	45.41 ± 0.62 ^b^	60.51 ± 1.60 ^d^	1.88 ± 0.13 ^d^	90.50 ± 2.39 ^d^	89.39 ± 1.31 ^d^
U_400_ EFA assembly	46.43 ± 0.13^c^	20.87 ± 0.26^c^	32.70 ± 0.22^c^	72.66 ± 1.31 ^c^	2.05 ± 0.17 ^c^	108.69 ± 2.06 ^c^	99.38 ± 1.45 ^c^
U_300_ EFA assembly	56.92 ± 0.71 ^b^	17.46 ± 1.00 ^d^	25.62 ± 0.70 ^d^	80.38 ± 1.63 ^b^	3.04 ± 0.25 ^b^	120.35 ± 2.44 ^b^	105.78 ± 1.34 ^b^
U_200_ EFA assembly	61.48 ± 0.28 ^a^	16.86 ± 0.58 ^de^	22.66 ± 0.51 ^e^	85.35 ± 1.97 ^a^	3.44 ± 0.53 ^a^	127.82 ± 2.45 ^a^	109.88 ± 1.44 ^a^

RDS, rapidly digestible starch; SDS, slowly digestible starch; RS, resistant starch; ***C*_∞_,** the equilibrium concentration of the digestive process; *k*, digestive kinetic rate constant; HI, hydrolysis index; EGI, estimated glycemic index. Samples labeled with different superscript letters in the same column differ significantly from one another (*p* < 0.05).

## Data Availability

The original contributions presented in this study are included in the article/Supplementary Material. Further inquiries can be directed to the corresponding authors.

## Supplementary Materials

The following supporting information can be downloaded at: https://www.mdpi.com/article/10.3390/foods15061021/s1, Figure S1: self-assembly characteristics of nano-scale EFA assemblies analyzed using 2D COS Total FTIR; Figure S2: total FTIR spectra of nano-scale EFA assemblies; Figure S3: AFM tip scanning trajectory along the cross-section of samples; Figure S4: correlation analysis between anti-digestibility and structure based on the Pearson correlation co-efficient; Table S1: chain length distribution of raw Euryale ferox starch and Euryale ferox amylopectin; Table S2: the nano-surface texture parameters of nano-scale EFA ternary assemblies; Table S3: the in vitro digestible characteristics of amylopectin ternary assemblies from staple crops pre-pared under high-power ultrasound.

## Abbreviations

WMA	White waxy maize amylopectin
SI	Self-assembly index
*R_c_*	Relative crystallinity
DP	Degree of polymerization
*CL*	Average degree of polymerization
*d*	Semicrystalline lamellae thickness
*d_a_*	Amorphous lamellae thickness
*d_c_*	Crystalline lamella thickness
*α*	Mass fractal exponent
*D_m_*	Mass fractal dimension
ξ	Average distance between crystalline and amorphous lamellae
*ξ* *_c_*	Crystallite unit thicknesses
PV	Peak viscosity
TV	Trough viscosity
BDV	Breakdown viscosity
FV	Final viscosity
SBV	Setback viscosity
PT	Pasting temperature
*M* *_n_*	Weight-average molecular weight
*M_w_*	Number-average molar mass
*R_g_*	Radius of gyration
PI	Polydispersity index (*M_w_*/*M_n_*)
*ν*	Conformation index
*ρ*	Molecular density
*T_o_*	Start temperature
*T_p_*	Gelatinization temperature
*T_c_*	Conclusion temperature
*R*	Temperature range
Δ*H_g_*	Gelatinization enthalpy
*Rq*	Nano root mean square roughness
RDS	Rapidly digestible starch
SDS	Slowly digestible starch
RS	Resistant starch
*C_∞_*	Equilibrium concentration
*k*	Speed rate constants
HI	Hydrolysis index
EGI	Estimated glycemic index
