# Supplementary material for: New Insight into the Multi-Scale Structure and Anti-Digestibility of Nano-Scale Amylopectin Ternary Assemblies Prepared Under High-Power Ultrasound
Source: Foods. 2026 Mar 14;15(6):1021. doi: 10.3390/foods15061021 (PMC13024885; doi:10.3390/foods15061021)
Supplement: Supplementary file 1 [file foods-15-01021-s001.zip › foods-4094535-supplementary.pdf]

Supplementary S1

Highlights

• Nano-scale *Euryale ferox* amylopectin assemblies were prepared under high-power ultrasound.

• Self-assembly sites within B2 and C chains increased as the ultrasound power increased.

• A compact and ordered molecular cross-linking network was observed after applying high-power ultrasound.

• The declined "blocklet" size caused an increase in resistant starch content and a more stable glycemic release with increasing ultrasound power.

• *Euryale ferox* assemblies showed higher anti-digestibility properties than amylopectin assemblies from staple crops, both of which were prepared under high-power ultrasound.

Supplementary S2

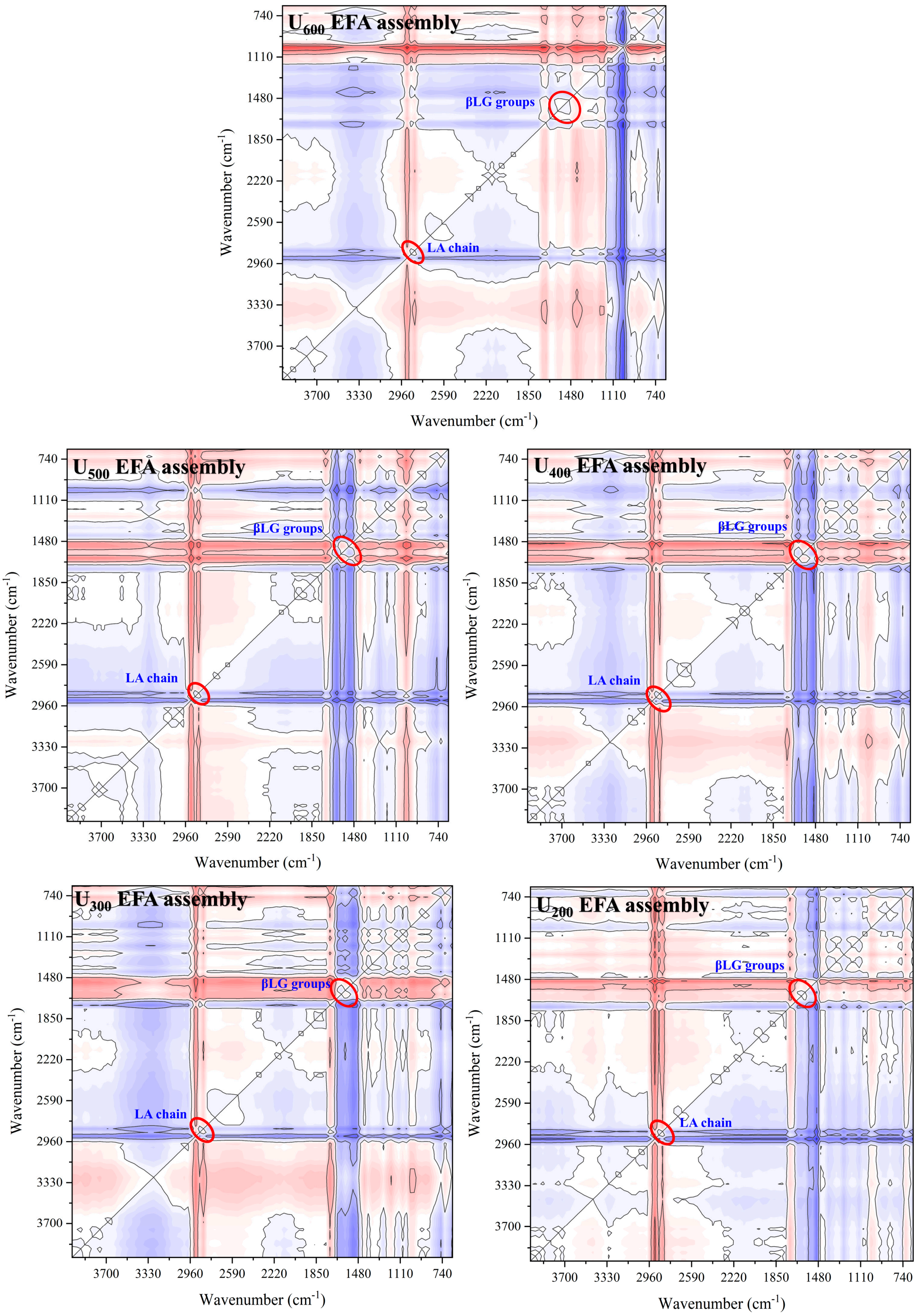

Figure S1. Self-assembly characteristics of nano-scale EFA assemblies analyzed using 2D COS Total FTIR.

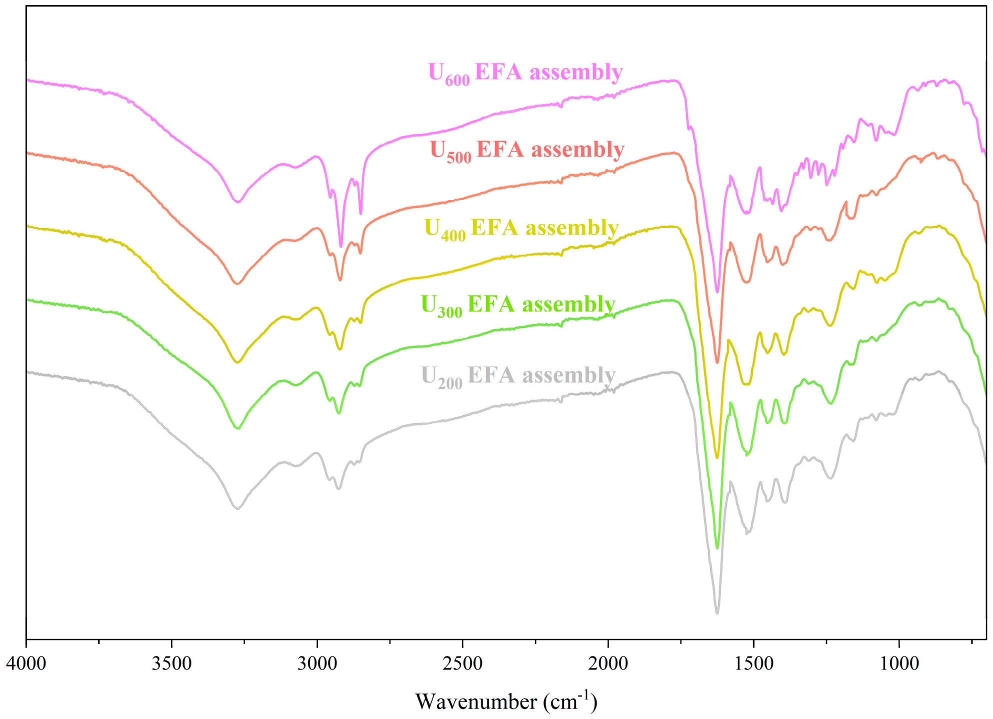

Figure S2. Total FTIR spectra of nano-scale EFA assemblies.

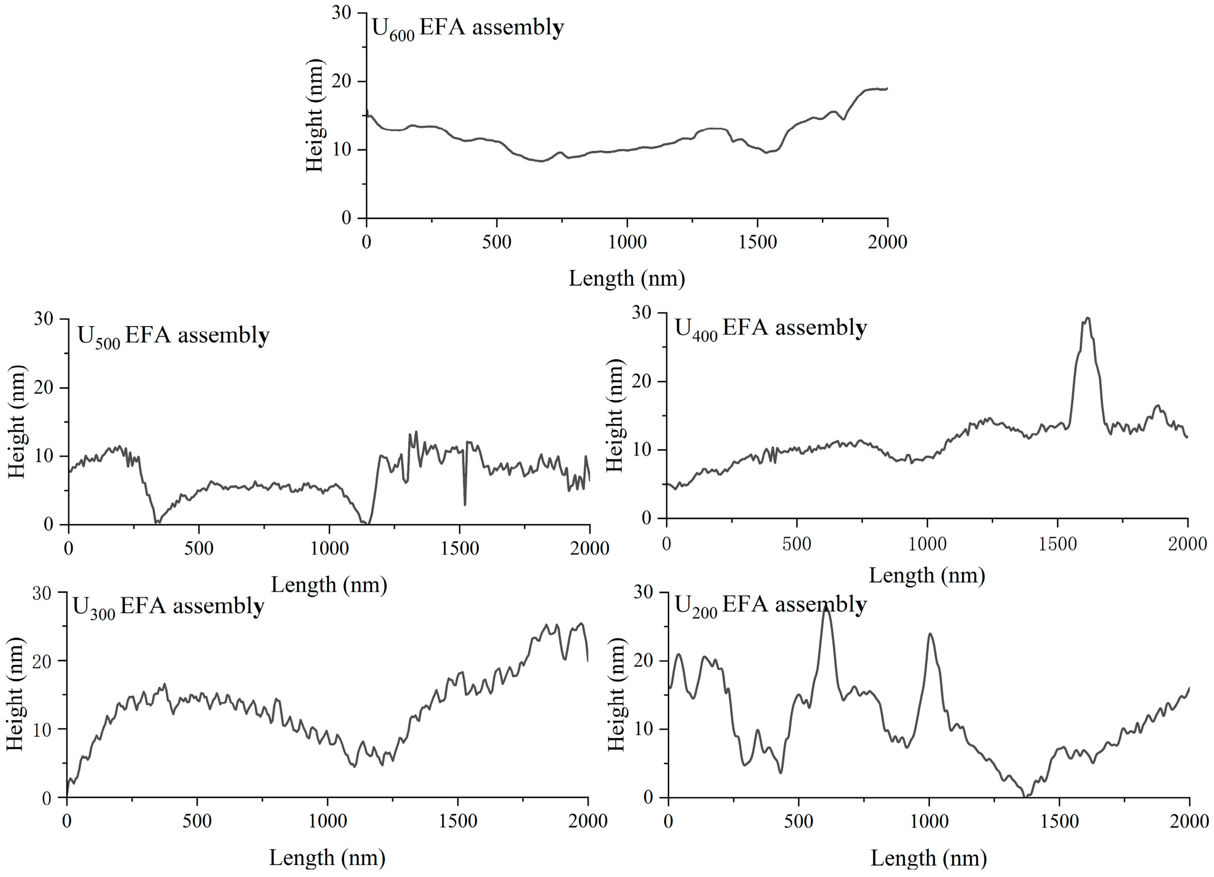

Figure S3. AFM tip scanning trajectory along the cross-section of samples.

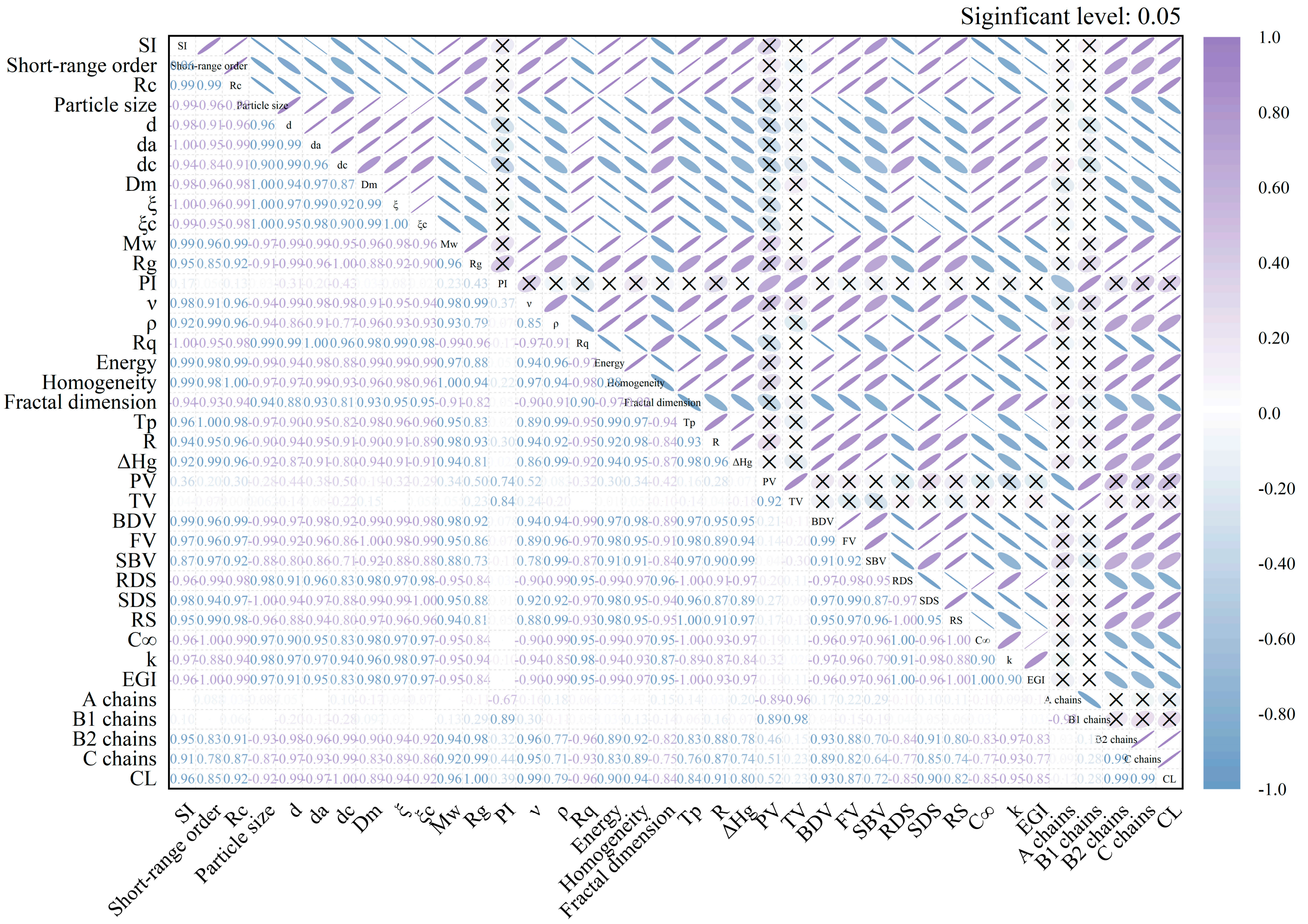

Figure S4. Correlation analysis between anti-digestibility and structure based on the *Pearson* correlation coefficient.

Table S1. Chain length distribution of raw *Euryale ferox* starch and *Euryale ferox* amylopectin.

**Starch sample**	A chains (DP = 6–12)	B1 chains (DP = 13–24)	B2 chains (DP = 25–36)	C chains (DP ≥ 37)	CL (DP)
Euryale ferox starch	19.76±0.17[a]	41.22±0.08[a]	19.05±0.20[a]	19.97±0.09[a]	22.57±0.16[a]
EFA	19.70±0.05[a]	41.26±0.22[a]	18.98±0.10[a]	20.06±0.20[a]	22.62±0.20[a]

Samples labeled with different superscript letters in the same column differ significantly from one another ($p < 0.05$).

Table S2. The nano-surface texture parameters of nano-scale EFA ternary assemblies.

R_q, nano root mean square roughness. Samples labeled with different superscript letters in the same column differ significantly from one another ($p < 0.05$).

Starch sample	R_q (nm)	Textural features				
		Energy (×10⁻³ J)	Contrast	Homogeneity	Entropy (e.u.)	Fractal dimension
U_{600} EFA assembly	$8.73±0.51^{e}$	$3.07±0.11^{a}$	$0.53±0.02^{de}$	$0.58±0.04^{a}$	$5.71±0.31^{e}$	$4.00±0.10^{de}$
U_{500} EFA assembly	$11.45±0.22^{d}$	$2.69±0.03^{b}$	$0.57±0.03^{d}$	$0.47±0.06^{b}$	$6.80±0.09^{d}$	$4.24±0.09^{d}$
U_{400} EFA assembly	$12.88±0.46^{c}$	$2.14±0.10^{c}$	$0.62±0.04^{c}$	$0.39±0.03^{c}$	$7.91±0.20^{c}$	$7.01±0.21^{c}$
U_{300} EFA assembly	$16.42±1.05^{b}$	$1.77±0.02^{d}$	$0.73±0.02^{b}$	$0.34±0.02^{cd}$	$9.09±0.14^{b}$	$7.55±0.19^{b}$
U_{200} EFA assembly	$19.69±0.71^{a}$	$1.50±0.05^{e}$	$0.88±0.02^{a}$	$0.21±0.02^{e}$	$11.24±0.16^{a}$	$8.14±0.17^{a}$

Table S3. The in vitro digestible characteristics of amylopectin ternary assemblies from staple crops prepared under high-power ultrasound.

Samples	RDS (%)	SDS (%)	RS (%)	C_∞	k	HI	EGI
U_{600} white waxy maize starch	$61.87±0.19^{d}$	$14.95±0.60^{a}$	$23.18±0.57^{a}$	$81.79± 1.07^{c}$	$3.17±0.15^{d}$	$122.69±1.61^{d}$	$107.05±0.88^{cd}$
U_{600} waxy potato starch	$70.44±0.09^{c}$	$8.87±0.63^{b}$	$20.69±0.62^{b}$	$84.12± 1.60^{b}$	$4.04±0.13^{c}$	$126.18±2.40^{abc}$	$108.97±1.32^{abc}$
U_{600} waxy wheat starch	$73.15±0.20^{b}$	$5.41±0.30^{c}$	$21.44± 0.22^{c}$	$84.95± 1.31^{b}$	$4.36 ±0.17^{b}$	$127.43±1.97^{ab}$	$109.66±1.08^{ab}$
U_{600} waxy rice starch	$76.52±1.03^{a}$	$4.33±1.25^{d}$	$19.15±0.70^{d}$	$87.19 ± 1.63^{a}$	$5.01 ±0.25^{a}$	$130.78±2.45^{a}$	$111.50±1.34^{a}$

RDS, rapidly digestible starch; SDS, slowly digestible starch; RS, resistant starch; C_∞, the equilibrium concentration of the digestive process; k, digestive kinetic rate constant; HI, hydrolysis index; EGI, estimated glycemic index. Samples labeled with different superscript letters in the same column differ significantly from one another ($p < 0.05$).

Amylopectin content analysis

A total of 200 mg of amylopectin was mixed with 1 mL of ethanol and 9 mL of 0.9 M NaOH and processed in a boiling water bath until complete dissolution. After cooling to 25 °C, the solution was diluted to a final volume of 100 mL with distilled water. An aliquot of 2.5 mL of the diluted solution was then further diluted to 50 mL. Subsequently, 1 mL of Lugol's iodine solution (saturated I_2–KI solution) was added, and the mixture was allowed to react for 20 min for color development. The absorbance was measured at 548 nm using a UV–Vis spectrophotometer, and the amylopectin content was determined from a standard curve constructed using an amylopectin standard [26].
